# SLC50A1 inhibits the doxorubicin sensitivity in hepatocellular carcinoma cells through regulating the tumor glycolysis

**DOI:** 10.1038/s41420-024-02261-3

**Published:** 2024-12-18

**Authors:** Ganggang Wang, Wenzhi Jin, Lianmei Zhang, Meiyuan Dong, Xin Zhang, Zhijie Zhou, Xiaoliang Wang

**Affiliations:** 1 Department of Hepatobiliary Surgery, Shanghai Pudong Hospital, Fudan University Pudong Medical Center, Shanghai, China; 2 Graduate School of Hebei Medical University, Shijiazhuang, People’s Republic of China; 3 Department of Endocrinology, Shanghai Pudong Hospital, Fudan University Pudong Medical Center, Shanghai, China

**Keywords:** Gene regulation, Cancer metabolism

## Abstract

Metabolic reprogramming has been found to be closely associated with the occurrence and development of hepatocellular carcinoma (HCC). The relationship between SLC50A1, a member of the SLC family involved in glucose transmembrane transport, and HCC remains unclear. This study aims to investigate the function and underlying mechanisms of SLC50A1 in the occurrence and progression of HCC. Based on bioinformatics analysis and clinical sample testing, we observed a significant upregulation of SLC50A1 in HCC, which is correlated with unfavorable prognosis in HCC patients. Additionally, there is a noticeable correlation between the expressions of SLC50A1 and METTL3. Further in vitro and in vivo experiments confirmed that SLC50A1 can regulate cellular glycolysis and the cell cycle, thereby promoting the proliferation of HCC cells while reducing apoptosis. Moreover, our findings indicate that SLC50A1 enhances resistance of HCC cells to DOX and 2-DG. Furthermore, we discovered that the m6A methyltransferase METTL3 mediates the methylation modification of SLC50A1. The recognition and binding of the modified SLC50A1 by IGF2BP2 subsequently promote its stability and translational expression. Consequently, our research identifies the METTL3/SLC50A1 axis as a novel therapeutic target in the context of HCC.

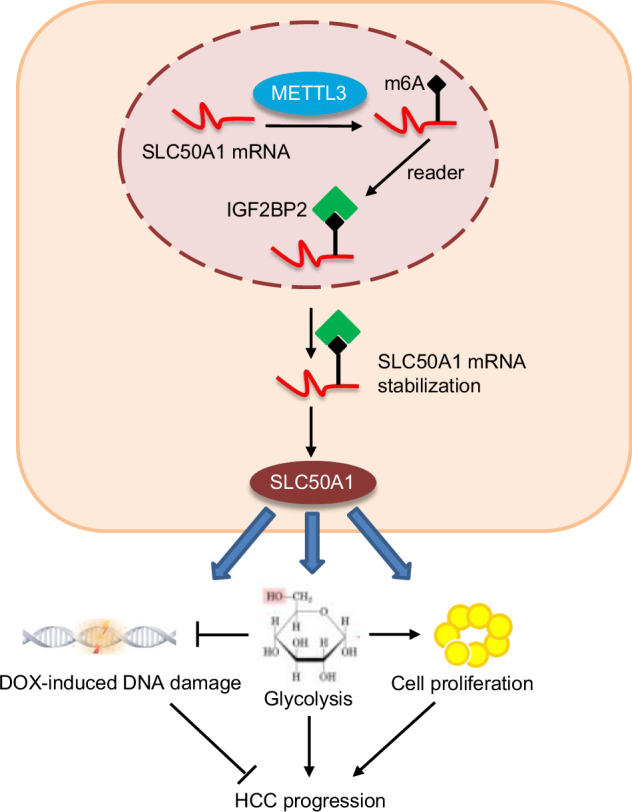

Hepatocellular carcinoma (HCC) stands as a prevalent malignancy on a global scale, characterized by elevated occurrence and considerable fatality rates. As per the most recent statistical data [1], in China, HCC ranks as the fourth most newly diagnosed cancer and is the second leading cause of cancer-related deaths. By 2030, there will be over a million patients who succumb to liver cancer, and the 5-year survival rate for HCC is merely 18%, making it the second most lethal cancer after pancreatic cancer [2]. Currently, surgical resection is the preferred treatment for hepatocellular carcinoma, but the majority of patients are not suitable candidates for surgery due to tumor size, location, and impaired liver function [3]. For patients with advanced and unresectable liver cancer, targeted therapy combined with monoclonal antibody treatment is a frontline therapeutic approach [4]. However, liver cancer still exhibits a high mortality rate. Therefore, there is a compelling need to delve deeper into the disease’s biology to explore novel treatment avenues [5].

Energy metabolism reprogramming, achieved by altering energy metabolism, is considered one of the hallmarks of cancer, promoting rapid growth and proliferation of cancer cells [6]. Metabolic reprogramming in liver cancer is of paramount significance for its initiation and progression. In recent years, strategies targeting the reprogramming of glucose metabolism have garnered extensive attention as potential treatments for liver cancer [7, 8].

Currently, the proteins known to mediate glucose transmembrane transport are primarily from the solute carrier (SLC) family, including SLC2, SLC5, and SLC50 families [9]. The SLC family is one of the largest membrane protein families encoded by the human genome, consisting of 65 families with a total of 400 members [10]. This family transports a diverse range of solutes, ranging from simple inorganic ions to amino acids, sugars, and even relatively complex organic molecules like hemoglobin. A newly discovered class of glucose transport proteins is the SWEET family. Human SWEET1 is encoded by the SLC50A1 gene, comprising 221 amino acids with a molecular weight of 25 kDa [9, 11]. Goat SWEET1 can activate AKT signaling, resulting in the high expression of GLUTI, GLUT4, and GLUT14 in goat mammary gland epithelial cells, and the DNA and amino acid sequences of goat SWEET1 are 85% and 83% identical to the sequences of human SWEET1, respectively [12]. Human SWEET1 is primarily found in the Golgi complex and serves as a component of the vesicular exocytosis pathway, participating in the efflux of glucose in the human intestinal and liver cells [13]. Previous research has revealed that SLC50A1 contributes to lactose synthesis in mammary glands by providing glucose [13]. Additionally, it has been identified as a potential serum diagnostic and prognostic marker for breast cancer [14, 15]. However, its association with liver cancer has not been reported in the existing literature.

N6-methyladenosine (m6A) is a unique chemical modification, and the m6A modification of mRNA has been found to be closely associated with various cancers, such as liver cancer [16], lung cancer [17], gastric cancer [18]. Through its ability to regulate various RNA metabolism processes, including stability, splicing, transcription, translation, and degradation, m6A plays a role in cancer cell proliferation and invasion [19–23]. Notably, m6A is strongly linked to drug resistance against various medications [24–27]. This suggests that modulating specific m6A-related genes or activating/inhibiting certain m6A regulatory factors could enhance tumor sensitivity to treatments, offering new avenues for drug development or combination therapies.

In this study, we identified through GEO and TCGA databases that SLC50A1 is significantly upregulated in liver cancer compared to normal tissues. Overexpression of SLC50A1 predicts unfavorable prognosis in liver cancer patients, a finding that was supported by our analysis of clinical tissues. Utilizing bioinformatics, in vitro cell experiments, and in vivo animal experiments, we found that SLC50A1 regulates cellular glycolysis and the cell cycle, promoting tumorigenesis. Moreover, we observed that SLC50A1 enhances the resistance of HCC cells to Doxorubicin (DOX) and 2-deoxy-D-glucose (2-DG), and it facilitates DNA damage repair in DOX-treated HCC cells through the glycolytic pathway. Additionally, we discovered that the m6A methyltransferase METTL3 mediates m6A modification of SLC50A1, which is recognized and bound by IGF2BP2, subsequently promoting the stability and translational expression of SLC50A1. Hence, our study identifies the METTL3/SLC50A1 axis as a novel relevant therapeutic target in the context of liver cancer.

## Result

### SLC50A1 is significantly upregulated in HCC and correlated with poor prognosis in patients

**Fig. 1:**
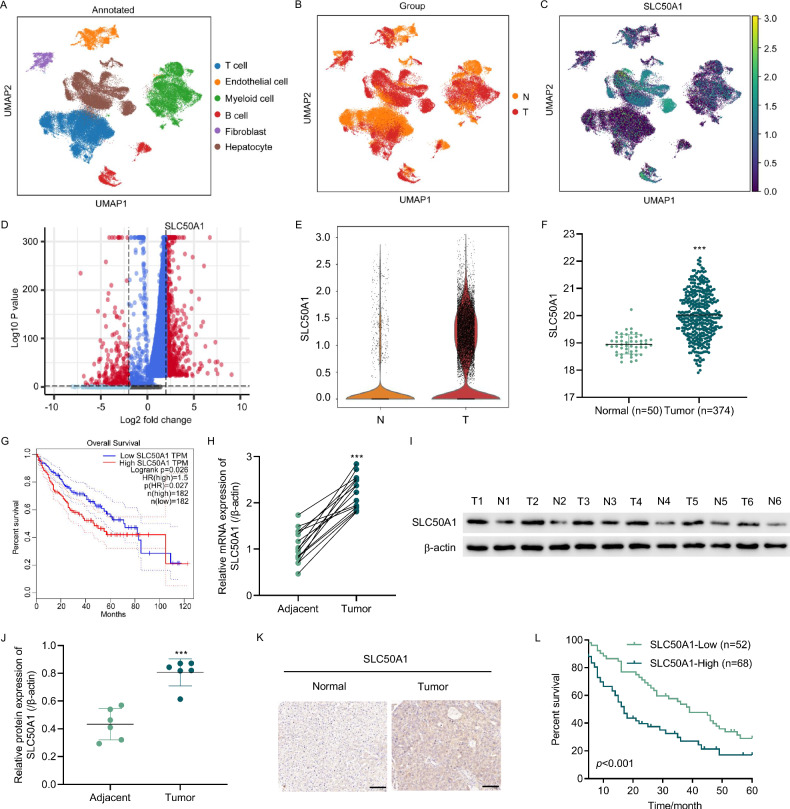
SLC50A1 is clinically relevant in HCC. **A** UMAP plot of all cells colored by each cell type in HCC patients (GSE149614). **B** UMAP plot showed the tumor cells and normal cells. **C** UMAP plot displayed the expression of SLC50A1. **D** Volcano plot showed the differentially expressed genes between tumor cells and normal cells. **E** Violin plot showed the expression of SLC50A1 in tumor cells and normal cells. **F** The expression of SLC50A1 in HCC and adjacent normal tissues in the TCGA dataset. **G** Survival analysis and comparison among people with high and low values of SLC50A1 expression in GEPIA database. Results of SLC50A1 (**H**) mRNA and (**I**, **J**) protein expression in HCC and the paired adjacent normal tissues of the hospital cohort. **K** Representative IHC images of SLC50A1 in HCC tissue microarrays. Scale bar: 100 μm. **L** Survival analysis and comparison among people with high and low expression of SLC50A1 expression in HCC tissue microarrays. ***P < 0.001 vs adjacent group.

We initiated our investigation by employing single-cell sequencing and bioinformatics analysis on the GSE149614 dataset. The results indicated a remarkable overexpression of SLC50A1 in liver cancer cell clusters (Fig. 1A–E). This finding was subsequently validated in the TCGA database (Fig. 1F). Further confirmation was obtained from the GEPIA database, illustrating a significant correlation between SLC50A1 expression and adverse prognosis in HCC patients (Fig. 1G). Simultaneously, we gathered clinical samples from 15 HCC patients. Our analysis unveiled a substantial increase in mRNA expression levels of SLC50A1 in HCC tissues compared to adjacent normal tissues (Fig. 1H). To deepen our understanding, we assessed the protein expression levels of SLC50A1 in 6 pairs of HCC tissues. Consistently, we found that SLC50A1 was significantly more expressed in tumor tissues than in normal tissues (Fig. 1I, J).

These accumulating results prompted our attention. Consequently, we conducted immunohistochemical staining on tissue microarrays from HCC patients. The outcomes clearly demonstrated a strong correlation between high SLC50A1 expression and poor prognosis in patients (Fig. 1K, L), as supported by clinical characteristics presented in Table 1. In summary, these findings collectively indicate a heightened expression of SLC50A1 in HCC and its association with unfavorable patient prognosis.

**Table 1:** Relationship between SLC50A1 expression and their clinic-pathological characteristics of HCC patients.

Characteristics	Cases	SLC50A1		*p*-value
		Low (*n* = 52)	High (*n* = 68)	
Age (years)				0.969
≥60	39	17	22	
<60	81	35	46	
Gender				0.828
Male	107	46	61	
Female	13	6	7	
Tumor size				
≥5 cm	89	32	57	0.006
<5 cm	31	20	11	
Differentiation grade				0.329
I–II	82	38	44	
III–IV	38	14	24	

Statistical analyses were performed by the Chi-square test.

### The downregulation of the SLC50A1 gene inhibits liver cancer cell growth by inducing G1 cell cycle arrest

To further investigate the expression of SLC50A1 in HCC cell lines, we compared the mRNA and protein expression levels of SLC50A1 in different HCC cell lines with normal liver cells (THLE-2). The results showed a significant increase in SLC50A1 expression in various HCC cell lines compared to THLE-2 cells (Fig. S1A–C).

**Fig. 2:**
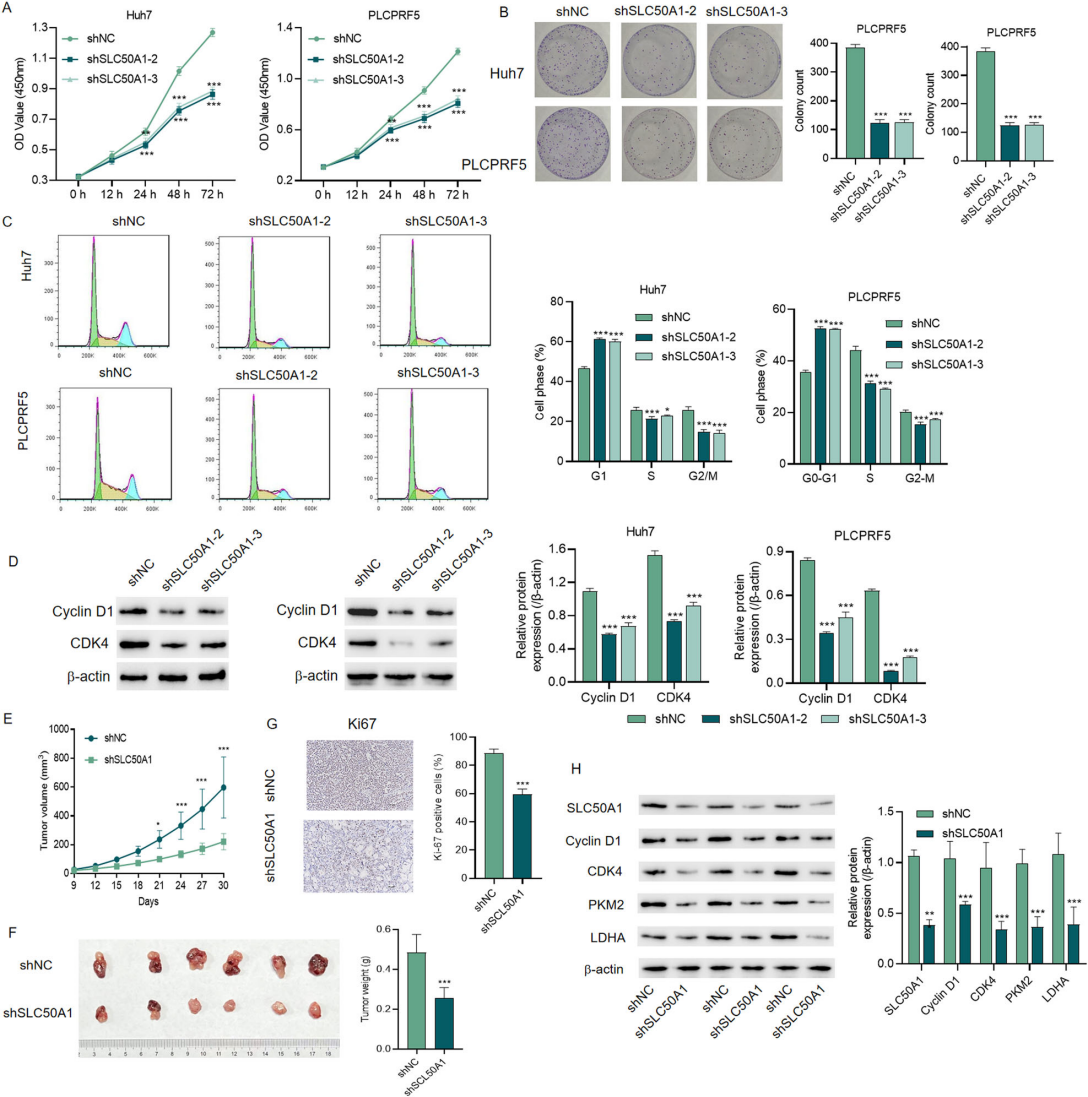
Knockdown of the SLC50A1 can inhibit the biological behavior and tumorigenic capacity of HCC cells. **A** Cell viability, (**B**) colony formation and (**C**) cell cycle, and (**D**) expression of Cyclin D1 and CDK4 of Huh7 and PLCPRF5 cells with or without SLC50A1 knockdown. **E** Tumor volume was monitored every 3 days for 30 days. **F** At day 30, mice were sacrificed, and tumors were photographed and weighed. **G** Ki67 IHC staining. **H** Expression of SLC50A1, Cyclin D1, CDK4, PKM2 and LDHA. Scale bar: 100 m. *P < 0.05, ***P < 0.001 vs shNC group.

To ascertain the impact of SLC50A1 on HCC, we performed knockdown experiments targeting SLC50A1 in Huh7 and PLCPRF5 cells. The efficiency of knockdown was validated (Fig. S2A, B), and the most effective shRNAs (shSLC50A1-2 and shSLC50A1-3) were chosen for subsequent investigations. Through CCK-8 and colony formation assays, we observed a significant reduction in cell proliferation and colony formation capacity upon downregulating SLC50A1 expression in Huh7 and PLCPRF5 cells (Fig. 2A, B). Consequently, we conducted cell cycle analysis on these SLC50A1-knockdown cell lines, revealing a substantial G1 cell cycle arrest in both Huh7 and PLCPRF5 cells compared to the control group (Fig. 2C). Further assessment of cell cycle-specific proteins Cyclin D1 and CDK4 demonstrated a marked decrease in their expression in SLC50A1-knockdown Huh7 and PLCPRF5 cells (Fig. 2D), consistent with previous flow cytometry results. These findings collectively suggest that the downregulation of the SLC50A1 gene inhibits liver cancer cell growth by inducing G1 cell cycle arrest.

This is consistent with our previous bioinformatics analysis. Through GSEA analysis, we found associations between SLC50A1 expression and several signaling pathways, including BENPORATH_PROLIFERATION, REACTOME_CELL_CYCLE, HALLMARK_GLYCOLYSIS, HAMAI_APOPTOSIS_VIA_TRAIL_UP, KANG_DOXORUBICIN_RESISTANCE_UP, and HALLMARK_DNA_REPAIR (Fig. S1D), which guided subsequent experimental studies.

Furthermore, to explore the in vivo growth of SLC50A1-knockdown HCC, we utilized a mouse xenograft model. The results were consistent with the in vitro experiments, as SLC50A1-knockdown HCC exhibited significantly slower growth in vivo (Fig. 2E, F). IHC and WB results similarly demonstrated that the expression of cell cycle-related proteins and metabolic-related proteins was significantly reduced in SLC50A1 knockout HCC tissues (Fig. 2G, H).

### SLC50A1 regulates HCC proliferation by affecting the glycolytic pathway

**Fig. 3:**
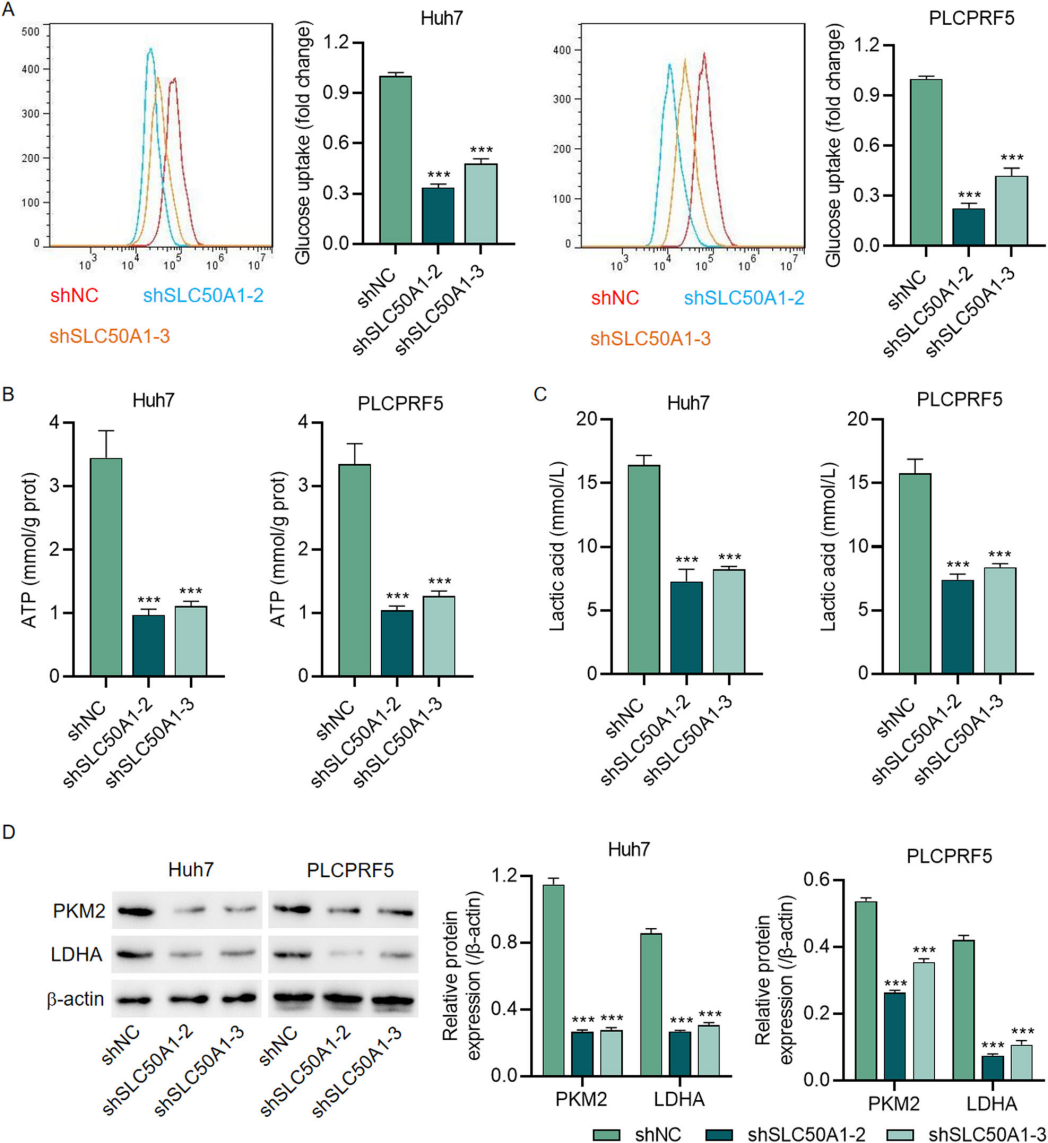
SLC50A1 knockdown inhibits glycolytic metabolism in HCC cells. **A** Glucose uptake, (**B**) ATP content, (**C**) lactate, and (**D**) expression of PKM2 and LDHA of Huh7 and PLCPRF5 cells with or without SLC50A1 knockdown. ***P < 0.001 vs shNC group.

As a glucose transporter, the relationship between SLC50A1 and glucose metabolism is integral. Therefore, we further investigated the influence of SLC50A1 expression on glycolysis. As depicted in Fig. 3A, downregulating SLC50A1 expression led to a noticeable reduction in glucose uptake in both Huh7 and PLCPRF5 cells. Biochemical analyses indicated that downregulation of SLC50A1 expression led to a simultaneous decrease in ATP and lactate levels in hepatocellular carcinoma cells (Fig. 3B, C). Additionally, the expression of PKM2 and LDHA was significantly reduced upon SLC50A1 knockdown (Fig. 3D). These findings indicate that SLC50A1 can impact glycolysis by influencing glucose uptake.

### 2-deoxy-D-glucose (2-DG) significantly inhibits the proliferation of liver cancer cells through the glycolytic pathway

Furthermore, we investigated the effects of 2-deoxy-D-glucose (2-DG), a known inhibitor of glycolysis, on the proliferation of liver cancer cells. Our findings revealed that treatment with 2-DG significantly inhibited the proliferation of liver cancer cells, suggesting that targeting glycolysis may be a viable strategy for limiting liver cancer growth. Collectively, these results indicate that SLC50A1 regulates HCC proliferation by influencing glucose uptake and glycolysis. Additionally, the inhibition of glycolysis by 2-DG highlights its potential as a therapeutic approach for liver cancer.

**Fig. 4:**
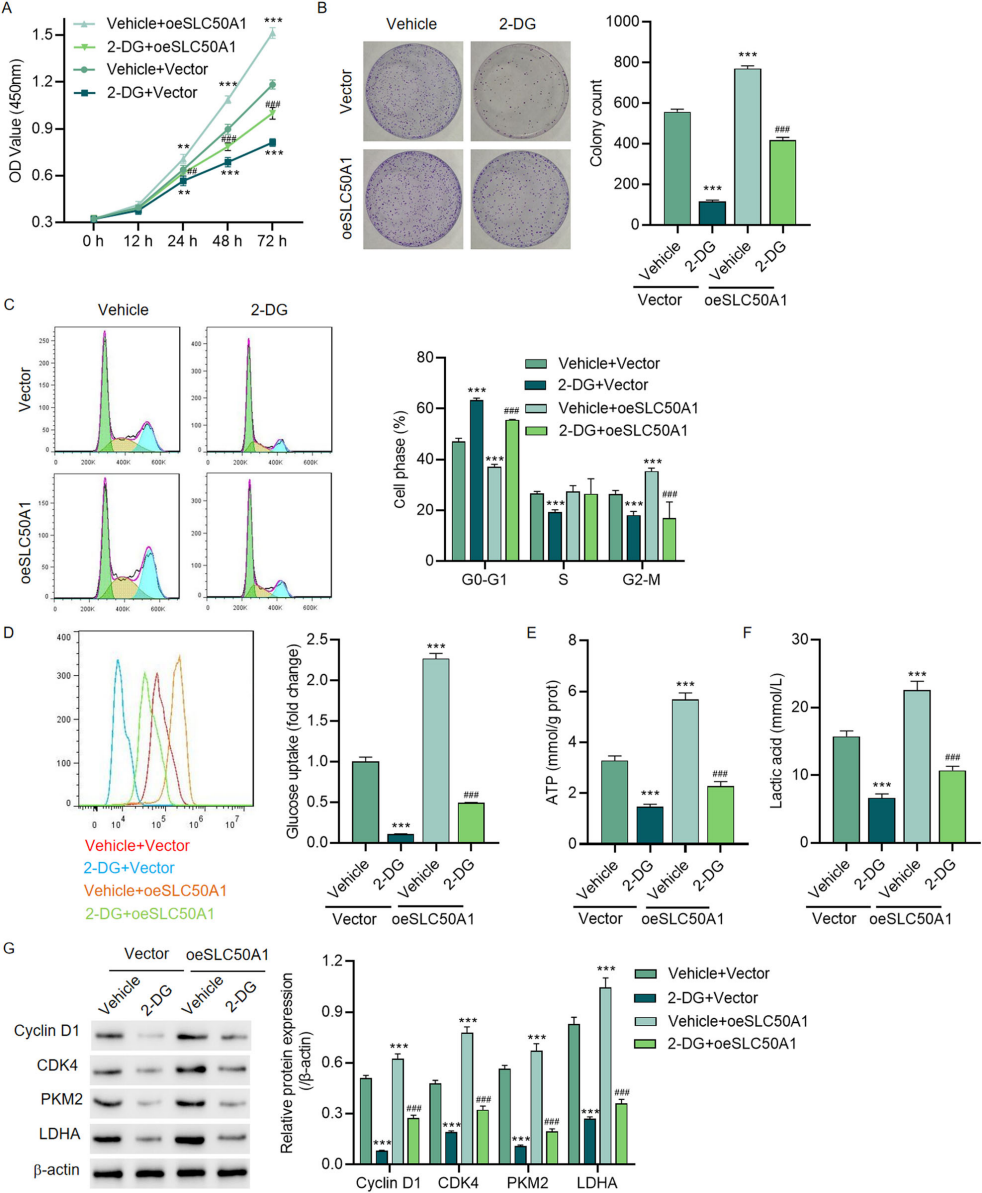
SLC50A1 overexpression promotes cell viability, colony formation, and cell cycle progression in HCC cells via the glycolysis pathway. **A** Cell viability, (**B**) colony formation, (**C**) cell cycle, (**D**) glucose uptake, (**E**) ATP content, (**F**) lactate, and (**G**) expression of Cyclin D1, CDK4, PKM2 and LDHA of HepG2 cells transduced with SLC50A1 expression vector and treated with 2-DG for 48 h. **P < 0.01, ***P < 0.001 vs Vehicle + Vector group. ^##^*P* < 0.01, ^###^*P* < 0.001 vs Vehicle+oeSLC50A1 group.

To further explore this, we selected HepG2 cells for SLC50A1 overexpression experiments. The transfection efficiency is depicted in Fig. S2C. CCK8 and colony formation assays showed that overexpression of SLC50A1 enhanced cell viability and proliferation in HepG2 cells (Fig. 4A, B). Cell cycle analysis indicated that SLC50A1 overexpression significantly reduced the percentage of cells in the G0-G1 phase while increasing the proportion of cells in the G2-M phase. This suggests that SLC50A1 overexpression stimulates rapid proliferation of hepatocellular carcinoma cells (Fig. 4C).

Interestingly, we also treated the cells with the glycolysis inhibitor 2-DG. Remarkably, despite SLC50A1 overexpression, 2-DG effectively inhibited the growth of HepG2 cells. However, HepG2 cells with SLC50A1 overexpression displayed enhanced resistance to 2-DG treatment (Fig. 4A–C). We conducted additional tests to measure glucose uptake, ATP, and lactate levels across various groups. The results showed that SLC50A1 overexpression significantly increased glucose uptake in HepG2 cells (Fig. 4D), leading to elevated ATP and lactate levels (Fig. 4E, F). Conversely, 2-DG effectively decreased glucose uptake, ATP, and lactate levels (Fig. 4D–F).

We further examined the expression of cell cycle-specific proteins Cyclin D1 and CDK4, as well as glycolysis-related proteins PKM2 and LDHA. The overexpression of SLC50A1 resulted in increased expression levels of Cyclin D1, CDK4, PKM2, and LDHA (Fig. 4G). In contrast, 2-DG treatment effectively reduced the expression of these proteins, while SLC50A1 overexpression enhanced HepG2 cells’ resistance to 2-DG (Fig. 4G).

Building on our earlier experiments that demonstrated the significant inhibition of liver cancer cell proliferation by 2-DG, we further explored its effects on tumor growth in vivo. As shown in Fig. S4, the administration of 2-DG markedly inhibited tumor growth in a subcutaneous xenograft mouse model (Fig. S3A, B). Further examinations revealed that 2-DG decreased the expression of Ki67, indicating reduced cell proliferation (Fig. S3C). Moreover, 2-DG treatment lowered the expression of Cyclin D1, CDK4, PKM2, and LDHA, without affecting SLC50A1 expression (Fig. S3D).

In conclusion, these results suggest that SLC50A1 modulates HCC proliferation by impacting the glycolytic pathway, and that 2-DG effectively inhibits liver cancer cell proliferation both in vitro and in vivo.

### METTL3 promotes m6A modification of SLC50A1 through the m6A reader IGF2BP2

**Fig. 5:**
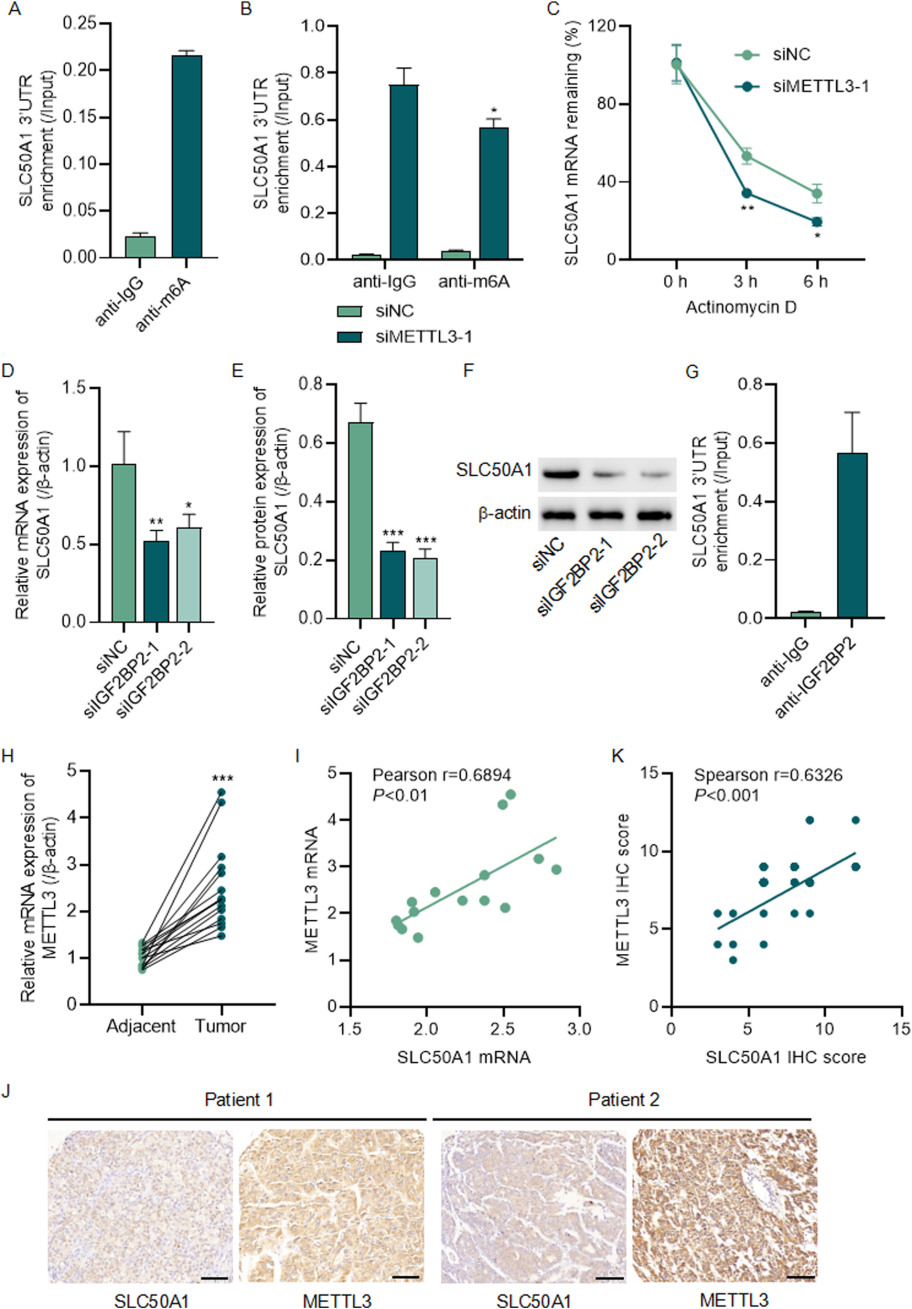
METTL3 promotes the m6A modification of SLC50A1 via the m6A reader IGF2BP2. **A** Relative m6A levels of SLC50A1 3’UTR in Huh7 cells were detected by RIP followed by RT-qPCR analysis. **B** SLC50A1 3’UTR enrichment in Huh7 cells transfected with METTL3 siRNA. **C** METTL3 knockdown in Huh7 cells decreased SLC50A1 mRNA stability. **D**–**F** SLC50A1 expression in Huh7 cells transfected with IGF2BP2 siRNA. **G** RIP followed by RT-qPCR assay was performed to examine the interaction between IGF2BP2 and SLC50A1 3’UTR. **H** Results of METTL3 mRNA expression in HCC and the paired adjacent normal tissues of the hospital cohort. **I** Correlation analysis between METTL3 and SLC50A1 mRNA expression. **J** Representative IHC images of SLC50A1 and METTL3 in HCC tissue microarrays. **K** Correlation analysis between METTL3 and SLC50A1 protein expression. Scale bar: 100 μm. *P < 0.05, **P < 0.01, ***P < 0.001 vs siNC group.

To further investigate the mechanism underlying the elevated expression of SLC50A1, we conducted RNA immunoprecipitation (RIP) followed by RT-qPCR analysis. This analysis revealed a significant increase in relative m6A levels within the SLC50A1 3’ UTR in liver cancer cells (Fig. 5A). Notably, this increased m6A enrichment was associated with METTL3. After knocking down METTL3 in Huh7 cells (Fig. S2D), we observed a marked decrease in the relative m6A levels within the SLC50A1 3’ UTR (Fig. 5B). Furthermore, METTL3 knockdown resulted in reduced mRNA stability of SLC50A1 (Fig. 5C). We then examined the role of downstream m6A readers, focusing on IGF2BP2. Upon knocking down IGF2BP2 expression in the cells (Fig. S2E), we noted a significant suppression of SLC50A1 expression (Fig. 5D, E, F). Additionally, RIP followed by RT-qPCR analysis revealed an interaction between the SLC50A1 3’ UTR and IGF2BP2 in Huh7 cells (Fig. 5G).

Finally, we investigated the relationship between METTL3 and SLC50A1 expression in clinical samples. Consistent with previous research, we found that METTL3 was significantly upregulated in liver cancer tissues. Importantly, we identified a significant correlation between the expression levels of METTL3 and SLC50A1 in HCC tissues (Fig. 5H, I, J, K). In summary, these findings indicate that METTL3 promotes the m6A modification of SLC50A1 through the m6A reader IGF2BP2. This interaction mechanism plays a crucial role in regulating SLC50A1 expression in liver cancer cells and may contribute to the pathogenesis of hepatocellular carcinoma.

### SLC50A1 regulates sensitivity of HCC cells to DOX

Doxorubicin (DOX) is a DNA topoisomerase II inhibitor widely used as a chemotherapy drug for various cancers, recognized as one of the most effective agents in clinical practice for treating malignant tumors [28]. Previous Gene Set Enrichment Analysis (GSEA) indicated a correlation between SLC50A1 expression and increased resistance to DOX (Fig. S1D).

**Fig. 6:**
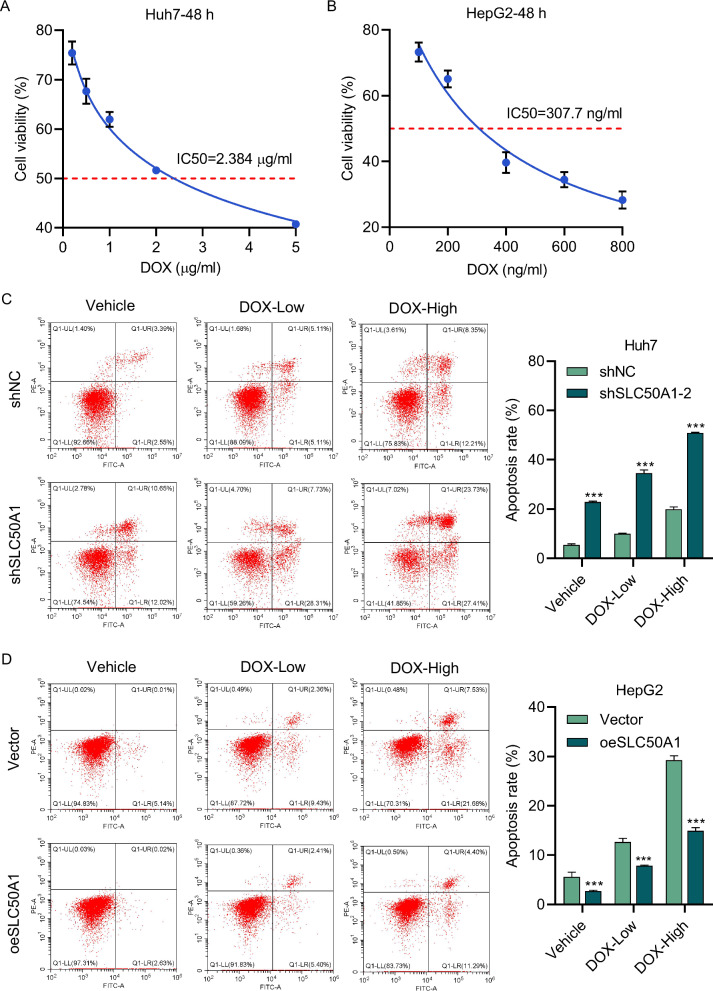
SLC50A1 regulates DOX sensitivity in HCC cells. **A** Huh7 cells were treated with different concentrations of DOX (0.2, 0.5, 1, 2, or 5 μg/ml) while (**B**) HepG2 cells were treated with different concentrations of DOX (100, 200, 400, 600, or 800 ng/ml), and cell viability was measured at 48 h. **C** Huh7 were transduced with SLC50A1 shRNA vector and treated with 0.2 (low) or 0.5 μg/ml (high) DOX while (**D**) HepG2 cells were transduced with SLC50A1 expression vector and treated with 100 (low) or 200 ng/ml (high) DOX, and cell apoptosis was measured at 48 h. ***P < 0.001 vs vector group.

In our study, we first assessed the viability of Huh7 and HepG2 cells at different DOX concentrations (Fig. 6A, B). We then treated the cells with selected DOX concentrations to evaluate apoptosis and investigate the relationship between SLC50A1 expression and DOX resistance. The results showed that Huh7 cells with downregulated SLC50A1 exhibited a significant increase in the apoptosis rate, while HepG2 cells overexpressing SLC50A1 displayed a decreased apoptosis rate (Fig. 6C, D).

These findings demonstrate that SLC50A1 expression modulates the sensitivity of HCC cells to DOX. To further validate this effect, we conducted in vivo experiments. The results indicated that knocking down SLC50A1 led to slower tumor growth and increased sensitivity to DOX, resulting in a reduced tumor growth rate (Fig. S4A, B). Additionally, TUNEL staining showed that downregulation of SLC50A1 enhanced apoptosis in tumor tissues (Fig. S4C).

### SLC50A1 modulates DNA damage repair and apoptosis in DOX-treated HCC cells through the glycolytic pathway

**Fig. 7:**
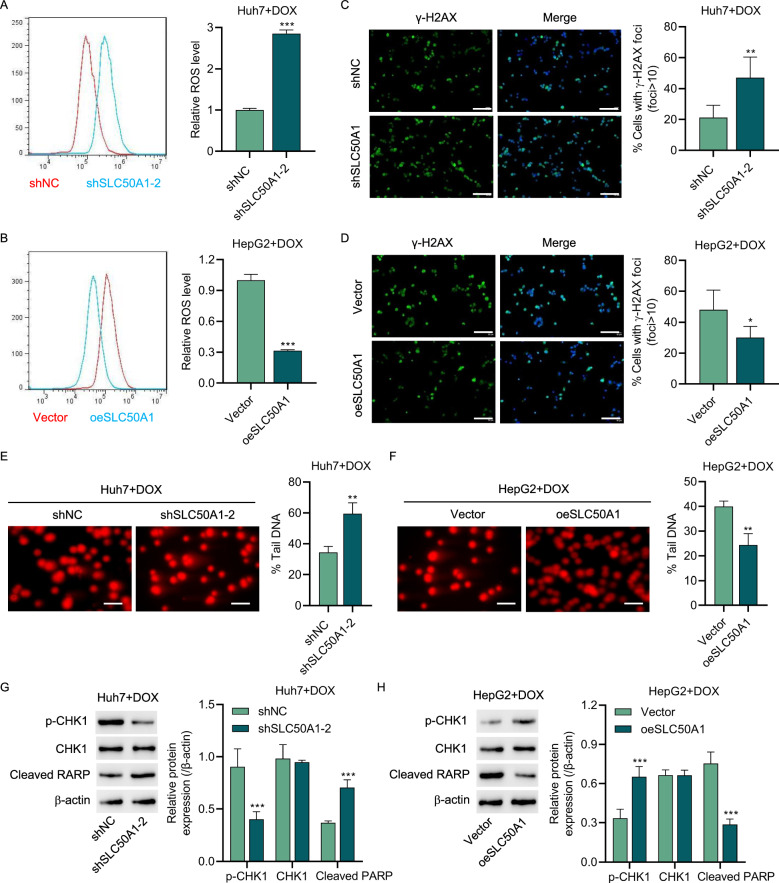
SLC50A1 regulates DNA damage repair in DOX-treated HCC cells. **C** Huh7 were transduced with SLC50A1 shRNA vector and treated with 0.5 μg/ml DOX while (**D**) HepG2 cells were transduced with SLC50A1 expression vector and treated with 200 ng/ml DOX, and (**A**, **B**) ROS production, (**C**, **D**) g-H2AX foci, (**E**, **F**) alkaline comets, and (**G**, **H**) expression of p-CHK1, CHK1, Cleaved RARP were measured. **C**, **D** Scale bar: 100 mm. **E**, **F** Scale bar: 50 μm. *P < 0.05, **P < 0.01, ***P < 0.001 vs shNC or vector group.

Previous GSEA results suggested a close association between SLC50A1 and DNA damage repair. In subsequent research, we found that under DOX treatment, Huh7 cells with downregulated SLC50A1 expression exhibited a significant increase in the production of reactive oxygen species (ROS), whereas HepG2 cells overexpressing SLC50A1 showed a noticeable decrease in ROS levels (Fig. 7A, B). This indicates that SLC50A1 expression can mitigate cell damage caused by oxidative stress by reducing ROS generation. Furthermore, γ-H2AX foci experiments revealed that downregulation of SLC50A1 led to an exacerbated degree of DNA double-strand breaks in DOX-treated Huh7 cells, while HepG2 cells overexpressing SLC50A1 displayed less DNA damage (Fig. 7C, D). These findings were corroborated by the alkaline comet assay, which showed that SLC50A1 overexpression reduced DOX-induced DNA damage in HCC cells (Fig. 7E, F). Moreover, our research indicated that SLC50A1 overexpression increased the expression of phosphorylated CHK1 (p-CHK1) while reducing the levels of cleaved PARP, suggesting that SLC50A1 aids in maintaining cell cycle stability, facilitating DNA damage repair, and subsequently reducing apoptosis.

**Fig. 8:**
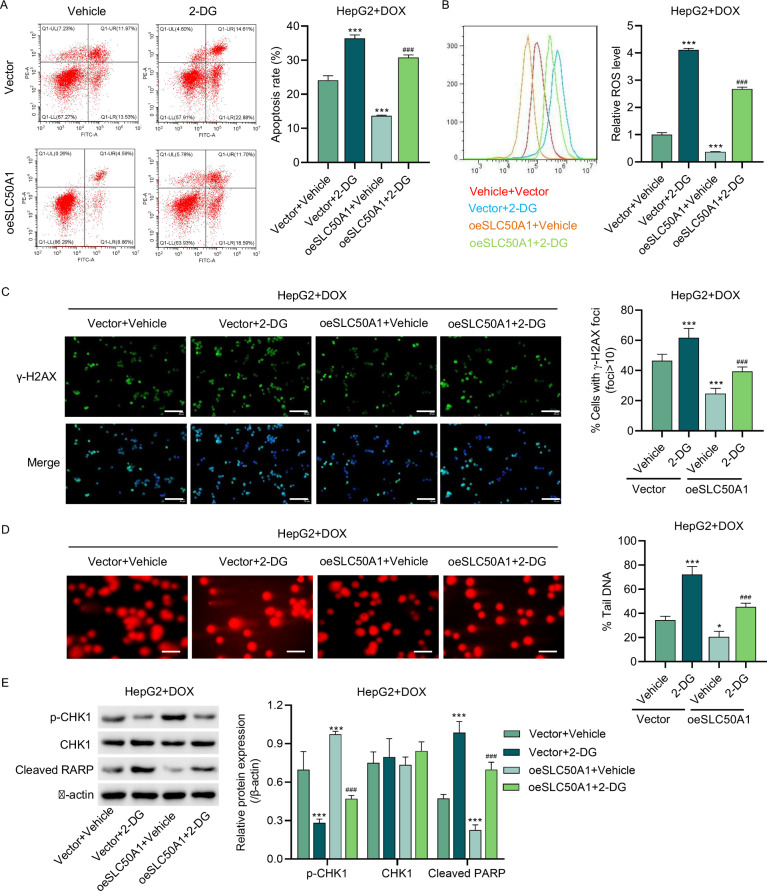
SLC50A1 overexpression promotes DNA damage repair in DOX-treated HCC cells via the glycolysis pathway. HepG2 cells were treated with 200 ng/ml DOX and transduced with SLC50A1 expression vector and/or treated with 2-DG, and the (**A**) cell apoptosis, (**B**) ROS production, (**C**) g-H2AX foci, (**D**) alkaline comets, and (**E**) expression of p-CHK1, CHK1, Cleaved RARP were measured. **C** Scale bar: 100 mm. **D** Scale bar: 50 μm. *P < 0.05, ***P < 0.001 vs shNC or Vehicle + Vector group. ^###^*P* < 0.001 vs oeSLC50A1 + Vehicle group.

In our study, we combined 2-deoxy-D-glucose (2-DG) treatment with HepG2 cells and observed that 2-DG further increased the apoptosis rate of HepG2 cells treated with DOX (Fig. 8A). Importantly, 2-DG was able to attenuate the DOX resistance conferred by SLC50A1 overexpression, suggesting that SLC50A1 may promote DOX resistance through the glycolytic pathway. We further investigated the effects of 2-DG on ROS production, γ-H2AX foci, and the alkaline comet assay. The results indicated that 2-DG exacerbated DNA damage in HepG2 cells, with changes in the expression of p-CHK1 and cleaved PARP supporting this observation (Fig. 8B–E).

In summary, these findings suggest that while SLC50A1 overexpression may enhance DNA damage repair in DOX-treated HCC cells via the glycolytic pathway, 2-DG can intensify cellular DNA damage.

## Discussion

Metabolic reprogramming not only enables cells to better adapt to the nutrient-deprived microenvironment but also contributes to the formation of specific immune barriers, leading to immune escape [29]. In certain cases, increased aerobic glycolysis can even promote the occurrence of epithelial-mesenchymal transition (EMT) in tumor cells, facilitating tumor metastasis [30–32]. For mammalian cells, glucose serves as the primary source of energy. Under aerobic conditions, complete glucose oxidation yields 36 ATP molecules from a single glucose molecule. However, under anaerobic conditions, glucose undergoes glycolysis, producing only two ATP molecules from one glucose molecule. Compared to normal cells, cancer cells exhibit a phenomenon known as the “Warburg effect,” wherein they preferentially undergo glycolysis even in the presence of oxygen [33, 34]. Consequently, cancer cells require increased glucose uptake, often accompanied by upregulated expression of glucose transporters. Glucose transporters play pivotal roles in the progression of various cancers, including pancreatic cancer [34, 35], gastric cancer [36], breast cancer [37], and cervical cancer [38]. Hence, glucose transporters hold great potential as therapeutic targets. Inhibiting glucose transporter expression could be a strategy to treat cancer patients. Currently, glucose transporters are employed in diagnostic techniques such as FDG-PET. Additionally, glucose transporters tagged with radioactive iodine isotopes offer promise for cancer treatment [39].

Numerous studies indicate disrupted glucose metabolism in the process of hepatocellular carcinoma (HCC) development. HCC cells exhibit distinct metabolic profiles from normal liver cells, often with aberrant expression of multiple metabolic enzymes, manifesting carcinogenic properties [40–44]. The solute carrier (SLC) transporter family comprises a group of transmembrane proteins that mediate the influx and efflux of solutes across plasma and intracellular membranes. They play a crucial role in drug resistance against anticancer agents [45]. Aberrant expression of various SLC family members has been associated with HCC progression, such as Solute carrier family 25 member 20 (SLC25A51) [46] and the roles of solute carrier family 2 member 9 (SLC2A9) [47]. In our study, we initially discovered significant upregulation of SLC50A1 in HCC. Furthermore, both bioinformatics analysis and clinical sample analysis indicated a correlation between SLC50A1 expression and poor patient prognosis. These findings suggest a potential oncogenic role for SLC50A1 in HCC. Our in vitro and in vivo experiments confirmed that downregulating SLC50A1 expression significantly reduces HCC cell proliferation and glycolytic capacity. Conversely, overexpression of SLC50A1 leads to a markedly increased malignant phenotype in HCC cells.

The Warburg effect has been found in liver cancer to be associated with the regulation of cell proliferation, immune evasion, invasion, metastasis, angiogenesis, and drug resistance [48–50]. In our study, we also observed that overexpression of SLC50A1 in HCC cells significantly increased glucose uptake, ATP production, and lactate levels. This indicates that SLC50A1 can promote glycolysis in HCC cells, enhancing the Warburg effect. 2-deoxy-D-glucose (2-DG), a glucose analog, competitively inhibits hexokinase 2 (HK2) and thus suppresses glycolysis, impacting HCC growth [51, 52]. It has been reported that the combination of conventional therapy and 2-DG synergistically inhibits the proliferation of sorafenib-sensitive and sorafenib-resistant HCC cells [53]. Moreover, we found that 2-DG can notably inhibit the malignant phenotype of HCC and suppress the growth of solid tumors in vivo. However, our findings suggest that SLC50A1 overexpression not only enhances glycolysis and proliferation in HCC cells while reducing apoptosis, but it also increases resistance to 2-DG.

Doxorubicin (DOX) has the capability to directly interact with cardiolipin situated on the inner mitochondrial membrane. This interaction enhances the generation of reactive oxygen species (ROS). Abundant intracellular ROS can cause significant harm to the structure of mitochondria, thereby inducing DNA damage through the impact of free radicals on DNA bases and sugar-phosphate backbones [54]. DNA damage that remains unaddressed can subsequently initiate processes such as cellular apoptosis, arrest of the cell cycle, and acceleration of cellular senescence [28, 55–57]. In our study, we observed that cells overexpressing SLC50A1 could repair DOX-induced DNA damage more quickly, thereby increasing HCC cells’ resistance to DOX. Conversely, DOX significantly increased apoptosis rates and induced DNA damage and ROS in SLC50A1-silenced HCC cells. However, in cells overexpressing SLC50A1, apoptosis rates and DNA chain damage were notably reduced. In vivo experiments similarly indicated that SLC50A1 expression affects tumor growth rates and DOX resistance, suggesting that SLC50A1 might facilitate DNA damage repair in DOX-treated HCC cells via the glycolytic pathway. Additionally, our combined treatment of 2-DG and DOX led to a significant increase in apoptosis rates in HCC cells, offering new avenues for HCC therapy.

m6A, as an epigenetic modification, has garnered increasing attention [58], and research has indicated the close relationship between RNA methylation and HCC metabolic reprogramming [59]. Methyltransferase-like 3 (METTL3), a protein possessing methyltransferase activity and affinity for S-adenosylmethionine, plays a pivotal role in the human methyltransferase complex. It governs the quantity and pattern of m6A modifications on the transcriptional level. Prior research has demonstrated a positive association between METTL3 expression and glucose metabolism in HCC [60]. Nevertheless, the direct influence of METTL3 on the m6A modification levels of critical glycolytic genes and the involvement of “reader” proteins in the m6A-dependent metabolic regulatory mechanism require more in-depth investigation.

In our study, we delved deeper into the upregulation mechanism of SLC50A1. We found that SLC50A1 undergoes m6A methylation, which is associated with METTL3 and insulin-like growth factor 2 mRNA binding protein 2 (IGF2BP2). Database and clinical microarray data correlation analyses also revealed a positive correlation between SLC50A1 and METTL3. IGF2BP2 is a significant RNA-binding protein and an m6A reader that enhances mRNA stability by recognizing and binding to m6A modification sites, thus regulating gene expression [61–64]. We observed that interfering with METTL3 or IGF2BP2 expression significantly reduced SLC50A1 expression. Furthermore, RIP-PCR results demonstrated that IGF2BP2 can bind to the SLC50A1 3’UTR, thereby stabilizing SLC50A1 expression. This suggests that METTL3 can promote SLC50A1 m6A modification, leading to its binding with IGF2BP2, and consequently, regulating SLC50A1 expression.

## Conclusion

Through bioinformatics, clinical specimens, and cell biology techniques, our study revealed the high expression of SLC50A1 in HCC, correlating with the malignant phenotype of HCC patients. The upregulation of SLC50A1 promotes HCC proliferation, reduces apoptosis, enhances resistance to DOX and 2-DG, and significantly increases the Warburg effect, thus controlling HCC cell metabolic reprogramming. Mechanistically, we found that the upregulation of SLC50A1 is attributed to its interaction with IGF2BP2 facilitated by METTL3. Our research identifies a novel target for HCC therapy and provides fresh insights into understanding HCC.

## Materials and methods

### Bioinformatics evaluation

We acquired single-cell RNA transcriptomic data from the GSE149614 dataset of liver cancer patients within the Gene Expression Omnibus (GEO) database, along with the raw RNA-seq expression counts sourced from HCC patient tissue samples within the TCGA HCC dataset. Through the utilization of Gene Set Enrichment Analysis (GSEA), we determined enriched pathways within the SLC50A1-high and SLC50A1-low groups. Additionally, we assessed the prognostic significance of SLC50A1 in HCC patients by leveraging the GEPIA database.

### Clinical samples

Fifteen surgically resected tumor samples, alongside their corresponding adjacent-normal samples, were procured from primary HCC patients hospitalized between January 2020 and July 2022. These samples were rapidly frozen in liquid nitrogen for preservation prior to analysis. Ethical approval for the study was obtained from the Hospital’s Ethics Committee, and all patients provided informed consent. To assess protein expression levels of SLC50A1 and METTL3, immunohistochemistry (IHC) was conducted using human HCC tissue microarrays (Shanghai Outdo Biotech, China). The tissue microarrays encompassed a total of 120 tumor tissue cases.

### Immunohistochemistry (IHC) procedure

Paraffin-embedded sections of primary HCC and adjacent normal tissues were prepared for IHC analysis. The specimens were subjected to staining with anti-SLC50A1 antibody (PA5-53721; Invitrogen, Thermo Fisher Scientific, Waltham, MA, USA) or anti-METTL3 antibody (ab195352; Abcam, Waltham, MA, USA). Subsequently, staining was visualized using HRP-conjugated anti-IgG antibody (D-3004; Long Island Biotech, China) in accordance with the standard protocol.

To quantify immunoreactivity, the H-score system was employed by two investigators. This involved assessing both the percentage of positively stained cells (graded on a scale of 0–4: 0, <5%; 1, 5–25%; 2, 25–50%; 3, 50–75%; 4, >75%) and the intensity of staining (graded on a scale of 0–3: 0, negative; 1, weak; 2, moderate; 3, strong). The combined scores ranged from 0 to 12. Based on the calculated immunoreactivity scores, the HCC patients were categorized into two groups: the low expression group (H-score < 6) and the high expression group (H-score ≥ 6).

### Cell culture

The human HCC cell lines (Hep3B, HuH7, PLCPRF5, and HepG2) and human healthy hepatocytes (THLE-2 cells) were procured from the Type Culture Collection of the Chinese Academy of Sciences (Shanghai, China) and the Cancer Institute and Hospital, Chinese Academy of Medical Sciences (CAMS) (Beijing, China). We regularly perform mycoplasma testing to ensure the absence of contamination. These cells were maintained in DMEM (Hyclone, USA) supplemented with 10% fetal bovine serum, and cultured in a CO_2_ incubator at 37 °C with 5% CO_2_. For experimental treatments, HepG2 cells were exposed to 25 mM 2-DG or various concentrations of DOX (100, 200, 400, 600, or 800 ng/ml), while HuH7 cells were treated with different DOX concentrations (0.2, 0.5, 1, 2, or 5 μg/ml), all for a duration of 48 h.

### Gene overexpression and knockdown

To target METTL3 or IGF2BP2, small interfering RNAs (siRNAs) from Shanghai GenePharma Co., Ltd were introduced into the HCC cells using DharmaFECT 1 siRNA infection reagent (Thermo Fisher Scientific). For SLC50A1 knockdown via RNA interference, short hairpin RNAs (shRNAs) were synthesized and cloned into a pLKO.1 vector (Addgene). To achieve SLC50A1 overexpression, the SLC50A1 gene was synthesized and inserted into pLVX-Puro vector (Clontech, USA). For generating recombinant plasmids, psPAX2 and pMD2G packaging vectors, a co-transfection approach was used in 293 T cells with Lipofectamine 2000 (Invitrogen, USA). Following transfection, virus particles were harvested after two days, concentrated through ultracentrifugation, and introduced into HCC cells via transduction. Virus particles obtained from cells transfected with pLKO.1-scramble shRNA (shNC) or empty pLVX-Puro vector served as negative controls.

### Cell viability assay

Cell viability was assessed using the Cell Counting Kit-8 (CCK-8) according to the manufacturer’s instructions. After 48 h of treatment, cells were plated in 96-well plates (3 × 10 ^ 3 cells/well) and exposed to a 10 mL CCK-8 solution for 1 h. The absorbance value (OD) at 450 nm was measured using a microplate reader to determine cell proliferation.

### Colony formation assay

Cells were seeded in 10 cm dishes 48 h post-treatment and cultured for two weeks. At the end of the incubation, colonies were fixed with paraformaldehyde for 15 min and stained with 0.5% crystal violet for 30 min. Colonies containing 50 cells or more were counted.

### Cell cycle assay

After 48 h of treatment, cells were centrifuged at 1000 × *g* for 5 min, fixed with 700 μL of pre-cooled absolute ethyl alcohol, incubated with 1 mg/mL RNase A (100 μL) in the dark for 30 min, and stained with 50 μg/mL propidium iodide (PI, 400 μL) for 10 min. FACScan flow cytometry using Cell Quest software was then performed.

### Measurement of glucose uptake

Glucose uptake was determined using a fluorescent glucose 2-NBDG (2-Deoxy-2-[(7-nitro-2,1,3-benzoxadiazol-4-yl) amino]-D-glucose) Uptake Assay Kit (BioVision, Milpitas, CA, USA) following the manufacturer’s protocol. Cells (5 × 10 ^ 5 cells per well in a six-well plate) were cultured for 24 h and starved of glucose for 3 h before treatment. After incubation with Krebs-Ringer Bicarbonate Buffer containing 2% bovine serum albumin for 40 min, 2-NBDG (100 µM) was added and incubated for 45 min at 37 °C. Cells were washed with PBS, trypsinized, resuspended in 10% FBS, and analyzed using a CytoFLEX flow cytometer.

### Measurement of lactate and ATP

After 48 h of treatment, cells (5 × 10 ^ 5 cells/well) were cultured in a six-well plate for one day at 37 °C. Lactate release and ATP content were determined using Lactic Acid assay kit and ATP assay kit, respectively, according to the manufacturer’s instructions.

### Analysis of reactive oxygen species (ROS)

ROS levels were assessed using flow cytometry and a ROS assay kit. After 48 h of treatment, cells were incubated with 10 µM 2′-7′dichlorofluorescin diacetate (DCFH-DA) dye for 20 min at 37 °C. Fluorescence intensity was measured using flow cytometry, and relative signal intensities of DCFH-DA were normalized to the control group.

### Cell apoptosis assay

Huh7 were collected 48 h after 0.2 or 0.5 μg/ml DOX treatment while HepG2 cells were collected 48 h after 100 or 200 ng/ml DOX treatment. The staining procedures followed two steps: (1) 20 min of incubation with Annexin-V-FITC at 4 °C and (2) another 20 min of incubation with propidium iodide (PI). Flow cytometer was then used to examine cell apoptosis.

### Immunofluorescence staining

Huh7 cells were plated and exposed to 0.5 μg/ml DOX, while HepG2 cells were plated and treated with 200 ng/ml DOX for a 48-hour incubation at 37 °C. Afterward, the plates were subjected to antibody staining against γ-H2AX (ab81299; Abcam, Cambridge, MA, USA), followed by DAPI staining. Imaging was carried out using a Zeiss fluorescence microscope (Carl Zeiss, Thornwood, NY), and quantification was performed using Bitplane Imaris 5.7 software (Bitplane AG, Zurich, Switzerland), following previously described methods [65].

### Comet assay

For the comet assay, Huh7 cells were treated with 0.5 μg/ml DOX, and HepG2 cells were treated with 200 ng/ml DOX for a 6-hour duration at 37 °C. The comet assay was subsequently conducted following established protocols [65].

### Quantitative RT-PCR (RT-qPCR)

Total RNA was extracted using Trizol Reagents and reverse transcribed into cDNA using the PrimeScript kit as per the manufacturer’s instructions. RT-qPCR was performed using the SYBR® Green kit on an ABI 7300 Real-Time PCR System (Applied Biosystem, USA). GAPDH was utilized as an internal control. Below are the primer sequences:

SLC50A1-F: 5ʹ-TCACCCTTGGCATGTTCTCC-3ʹ,

SLC50A1-R: 5ʹ-AGGAGAAGGACCCCTAGCAG-3ʹ;

METTL3-F: 5ʹ-CCCTATGGGACCCTGACAGA-3ʹ,

METTL3-R: 5ʹ-CTGGTTGAAGCCTTGGGGAT-3ʹ;

IGF2BP2-F: 5ʹ-GACAGGTCCTGCTGAAGTCC-3ʹ,

IGF2BP2-R: 5ʹ-TGTTGACTTGTTCCACATTCTCC-3ʹ;

b-actin-F: 5ʹ-AGGATTCCTATGTGGGCGAC-3ʹ,

b-actin-R: 5ʹ-ATAGCACAGCCTGGATAGCAA-3ʹ.

The relative mRNA expression was calculated by the 2^−ΔΔCT^ method.

### Western blotting

Whole-cell lysates were prepared using Radioimmunoprecipitation Assay (RIPA) lysis buffer supplemented with a protease inhibitor cocktail (Sigma, USA). The lysates were separated using SDS-PAGE gels and subsequently transferred to nitrocellulose membranes (Millipore, Bedford, USA). After blocking, the membranes were incubated with specific primary antibodies against SLC50A1 (PA5-70596; Invitrogen, Thermo Fisher Scientific), PKM2 (4053; Cell Signaling Technology, Danvers, MA, USA), LDHA (3582; Cell Signaling Technology), Cyclin D1 (55506; Cell Signaling Technology), CDK4 (12790; Cell Signaling Technology), METTL3 (ab195352; Abcam), IGF2BP2 (ab124930; Abcam), CHK1 (ab32531; Abcam), p-CHK1 (ab79758; Abcam), Cleaved PARP1 (ab32064; Abcam) and β-actin (60066-1-AP; Proteintech Group, Inc., Rosemont, IL, USA). After washing, the membranes were probed with the HRP-conjugated secondary antibody (AB-2301, ZB-2305; ZSGB-BIO, Beijing, China). Finally, the membranes were visualized using the enhanced chemiluminescence system (Bio-Rad, USA).

### Analysis of m^6^A content

Total RNA was extracted using Trizol reagent (Invitrogen). Poly(A) + RNA was purified using the GenEluteTM mRNA Miniprep Kit (Sigma, Louis, MO, USA). The m6A content in the total RNA was assessed using the m6A RNA Methylation Assay Kit (ab185912; Abcam). In brief, 80 µL of binding solution and 200 ng of sample RNA were added to each designated well, followed by incubation at 37 °C for 90 min for RNA binding. After washing each well three times with wash buffer, 50 µL of diluted capture antibody was added and incubated at room temperature for 60 min. Subsequently, each well was incubated with detection antibody and enhancer solution at room temperature for 30 min. Finally, the wells were exposed to developer solution in the dark for 1 to 10 min at 25 °C. The reaction was halted with stop solution and measured using a microplate reader at a wavelength of 450 nm within 2 to 10 min.

### RNA immunoprecipitation assays

RNA immunoprecipitation (RIP) assays were conducted using the Magna RIP RNA-Binding Protein Immunoprecipitation kit (Millipore). Cells were lysed using RIP lysis buffer, and the RNA-protein complexes were incubated with anti-m6A (ab208577; Abcam), anti-IGF2BP2 (ab128175; Abcam), or anti-IgG antibody (ab172730; Abcam) overnight at 4 °C. Afterward, the complexes were washed with RIP-wash buffer for 10 min at 4 °C, followed by washes with RIP-lysis buffer for 5 min at 4 °C. The co-precipitated RNAs were purified using phenol:chloroform:isoamyl alcohol and subjected to RT-qPCR.

### mRNA stability measurement

Cells were treated with 0.2 mM actinomycin D for 30 min, and the samples collected at 0 h were used as baseline. Additional samples were collected at 3 and 6 h after treatment, and total RNA was extracted from these samples. Reverse transcription using oligo(dT) primers was performed to synthesize cDNA. Quantification of mRNA levels was carried out through RT-qPCR.

### In vivo tumor xenograft model

Male nude mice aged 4 to 6 weeks were obtained from the Shanghai Laboratory Animal Company. All experimental procedures were conducted following the animal ethics guidelines and protocols approved by the Pudong Hospital. Mice were randomly assigned to experimental groups for subcutaneous injections. Each group received injections of 5 × 10^6^ Huh7 cells, transduced with or without the SLC50A1 shRNA vector, or 5 × 10^6^ HepG2 cells, administered in the armpit region. Treatment with a glycolysis inhibitor, 2-deoxy-d-glucose (2-DG) (250 mg/kg/day), or DOX (5 mg/kg, administered every three days), began on the 9th day post-injection. Tumor size was monitored every three days, and on day 30, tumors were excised from euthanized mice, measured for size, weighed, and photographed. To assess proliferation and apoptosis, sections were subjected to Ki67 IHC staining using an anti-Ki67 antibody (ab15580; Abcam) or terminal-deoxynucleotidyl transferase-mediated nick end labeling (TUNEL; Sigma, Shanghai, China) staining.

### Statistical analysis

All experiments were independently repeated at least three times. Data were presented as mean ± standard deviation (SD). Statistical analyses were performed using GraphPad Prism 8.4.2. Comparison between different experimental groups was conducted using the student *t*-test and ANOVA. Overall survival rate was calculated using the Kaplan-Meier method and Cox’s proportional hazards regression model. The log-rank test was applied to determine statistical differences between groups. A significance level of *P* < 0.05 was considered statistically significant.

## Supplementary information

SUPPLEMENTAL MATERIAL

Original Data File

## Data Availability

The data that support the findings of this study are available on request from the corresponding author.

## References

[1] Zheng R, Zhang S, Zeng H, Wang S, Sun K, Chen R, et al. Cancer incidence and mortality in China, 2016. J Natl Cancer Cent. 2022;2:1–9. 39035212 10.1016/j.jncc.2022.02.002 PMC11256658

[2] Jemal A, Ward EM, Johnson CJ, Cronin KA, Ma J, Ryerson B, et al. Annual report to the nation on the status of cancer, 1975–2014, featuring survival. J Natl Cancer Inst. 2017;109:djx030. 28376154 10.1093/jnci/djx030 PMC5409140

[3] Llovet JM, Fuster J, Bruix J. The Barcelona approach: diagnosis, staging, and treatment of hepatocellular carcinoma. Liver Transplant Off Publ Am Assoc Study Liver Dis Int Liver Transplant Soc. 2004;10:S115–20. 10.1002/lt.20034 14762851

[4] Finn RS, Qin S, Ikeda M, Galle PR, Ducreux M, Kim TY, et al. Atezolizumab plus Bevacizumab in Unresectable Hepatocellular Carcinoma. N. Engl J Med. 2020;382:1894–905. 32402160 10.1056/NEJMoa1915745

[5] Leslie J, Hunter JE, Collins A, Rushton A, Russell LG, Ramon-Gil E, et al. c-Rel-dependent Chk2 signaling regulates the DNA damage response limiting hepatocarcinogenesis. Hepatology. 2023;78:1050–63. 10.1002/hep.32781 PMC10521790 36089330

[6] Callao V, Montoya E. Toxohormone-like factor from microorganisms with impaired respiration. Science. 1961;134:2041–2. 13875778 10.1126/science.134.3495.2041

[7] Ye Y, Wang M, Wang G, Mai Z, Zhou B, Han Y, et al. lncRNA miR4458HG modulates hepatocellular carcinoma progression by activating m6A-dependent glycolysis and promoting the polarization of tumor-associated macrophages. Cell Mol Life Sci CMLS. 2023;80:99. 36933158 10.1007/s00018-023-04741-8 PMC11072995

[8] Zheng J, Yan X, Lu T, Song W, Li Y, Liang J, et al. CircFOXK2 promotes hepatocellular carcinoma progression and leads to a poor clinical prognosis via regulating the Warburg effect. J Exp Clin Cancer Res CR. 2023;42:63. 36922872 10.1186/s13046-023-02624-1 PMC10018916

[9] Wright EM. Glucose transport families SLC5 and SLC50. Mol Asp Med. 2013;34:183–96. 10.1016/j.mam.2012.11.002 23506865

[10] Alexander SPH, Kelly E, Mathie A, Peters JA, Veale EL, Armstrong JF, et al. The concise guide to pharmacology 2019/20: transporters. Br J Pharmacol. 2019;176:S397–493. 31710713 10.1111/bph.14753 PMC6844579

[11] Pliszka M, Szablewski L. Glucose transporters as a target for anticancer therapy. Cancers. 2021;13:4184. 34439338 10.3390/cancers13164184 PMC8394807

[12] Zhu LQ, Bao ZK, Hu WW, Lin J, Yang Q, Yu QH. Cloning and functional analysis of goat SWEET1. Genet Mol Res GMR. 2015;14:17124–33. 26681059 10.4238/2015.December.16.12

[13] Chen LQ, Hou BH, Lalonde S, Takanaga H, Hartung ML, Qu XQ, et al. Sugar transporters for intercellular exchange and nutrition of pathogens. Nature. 2010;468:527–32. 21107422 10.1038/nature09606 PMC3000469

[14] Zhang Q, Fang Y, She C, Zheng R, Hong C, Chen C, et al. Diagnostic and prognostic significance of SLC50A1 expression in patients with primary early breast cancer. Exp Ther Med. 2022;24:616. 36160901 10.3892/etm.2022.11553 PMC9468843

[15] Wang Y, Shu Y, Gu C, Fan Y. The novel sugar transporter SLC50A1 as a potential serum-based diagnostic and prognostic biomarker for breast cancer. Cancer Manag Res. 2019;11:865–76. 30697078 10.2147/CMAR.S190591 PMC6340503

[16] Zhang C, Huang S, Zhuang H, Ruan S, Zhou Z, Huang K, et al. YTHDF2 promotes the liver cancer stem cell phenotype and cancer metastasis by regulating OCT4 expression via m6A RNA methylation. Oncogene. 2020;39:4507–18. 32366907 10.1038/s41388-020-1303-7

[17] Yin H, Chen L, Piao S, Wang Y, Li Z, Lin Y, et al. M6A RNA methylation-mediated RMRP stability renders proliferation and progression of non-small cell lung cancer through regulating TGFBR1/SMAD2/SMAD3 pathway. Cell Death Differ. 2023;30:605–17. 34628486 10.1038/s41418-021-00888-8 PMC9984538

[18] Xu W, Lai Y, Pan Y, Tan M, Ma Y, Sheng H, et al. m6A RNA methylation-mediated NDUFA4 promotes cell proliferation and metabolism in gastric cancer. Cell Death Dis. 2022;13:715. 35977935 10.1038/s41419-022-05132-w PMC9385701

[19] Sendinc E, Shi Y. RNA m6A methylation across the transcriptome. Mol Cell. 2023;83:428–41. 36736310 10.1016/j.molcel.2023.01.006

[20] Zhuang H, Yu B, Tao D, Xu X, Xu Y, Wang J, et al. The role of m6A methylation in therapy resistance in cancer. Mol Cancer. 2023;22:91. 37264402 10.1186/s12943-023-01782-2 PMC10233906

[21] Chen M, Wong CM. The emerging roles of N6-methyladenosine (m6A) deregulation in liver carcinogenesis. Mol Cancer. 2020;19:44. 32111216 10.1186/s12943-020-01172-y PMC7047367

[22] Yang B, Wang JQ, Tan Y, Yuan R, Chen ZS, Zou C. RNA methylation and cancer treatment. Pharmacol Res. 2021;174:105937. 34648969 10.1016/j.phrs.2021.105937

[23] Zhao BS, Roundtree IA, He C. Post-transcriptional gene regulation by mRNA modifications. Nat Rev Mol Cell Biol. 2017;18:31–42. 27808276 10.1038/nrm.2016.132 PMC5167638

[24] Yu H, Yang X, Tang J, Si S, Zhou Z, Lu J, et al. ALKBH5 inhibited cell proliferation and sensitized bladder cancer cells to cisplatin by m6A-CK2α-mediated glycolysis. Mol Ther Nucleic Acids. 2021;23:27–41. 33376625 10.1016/j.omtn.2020.10.031 PMC7744648

[25] Taketo K, Konno M, Asai A, Koseki J, Toratani M, Satoh T, et al. The epitranscriptome m6A writer METTL3 promotes chemo- and radioresistance in pancreatic cancer cells. Int J Oncol. 2018;52:621–9. 29345285 10.3892/ijo.2017.4219

[26] Tang B, Yang Y, Kang M, Wang Y, Wang Y, Bi Y, et al. m(6)A demethylase ALKBH5 inhibits pancreatic cancer tumorigenesis by decreasing WIF-1 RNA methylation and mediating Wnt signaling. Mol Cancer. 2020;19:3. 31906946 10.1186/s12943-019-1128-6 PMC6943907

[27] Li K, Peng ZY, Gao S, Wang QS, Wang R, Li X, et al. M6A associated TSUC7 inhibition contributed to Erlotinib resistance in lung adenocarcinoma through a notch signaling activation dependent way. J Exp Clin Cancer Res CR. 2021;40:325. 34656164 10.1186/s13046-021-02137-9 PMC8520306

[28] Kciuk M, Gielecińska A, Mujwar S, Kołat D, Kałuzińska-Kołat Ż, Celik I, et al. Doxorubicin-an agent with multiple mechanisms of anticancer activity. Cells. 2023;12:659. 36831326 10.3390/cells12040659 PMC9954613

[29] Xia H, Huang Z, Xu Y, Yam JWP, Cui Y. Reprogramming of central carbon metabolism in hepatocellular carcinoma. Biomed Pharmacother Biomed Pharmacother. 2022;153:113485. 36076503 10.1016/j.biopha.2022.113485

[30] Yang J, Ren B, Yang G, Wang H, Chen G, You L, et al. The enhancement of glycolysis regulates pancreatic cancer metastasis. Cell Mol Life Sci CMLS. 2020;77:305–21. 31432232 10.1007/s00018-019-03278-z PMC11104916

[31] Kamarajugadda S, Stemboroski L, Cai Q, Simpson NE, Nayak S, Tan M, et al. Glucose oxidation modulates anoikis and tumor metastasis. Mol Cell Biol. 2012;32:1893–907. 22431524 10.1128/MCB.06248-11 PMC3347404

[32] Lu J, Tan M, Cai Q. The Warburg effect in tumor progression: mitochondrial oxidative metabolism as an anti-metastasis mechanism. Cancer Lett. 2015;356:156–64. 24732809 10.1016/j.canlet.2014.04.001 PMC4195816

[33] Warburg O. The chemical constitution of respiration ferment. Science. 1928;68:437–43. 17782077 10.1126/science.68.1767.437

[34] Warburg O. On the origin of cancer cells. Science. 1956;123:309–14. 13298683 10.1126/science.123.3191.309

[35] Lemstrová R, Souček P, Melichar B, Mohelnikova-Duchonova B. Role of solute carrier transporters in pancreatic cancer: a review. Pharmacogenomics. 2014;15:1133–45. 25084206 10.2217/pgs.14.80

[36] Xie J, Zhu XY, Liu LM, Meng ZQ. Solute carrier transporters: potential targets for digestive system neoplasms. Cancer Manag Res. 2018;10:153–66. 29416375 10.2147/CMAR.S152951 PMC5788932

[37] Panda S, Banerjee N, Chatterjee S. Solute carrier proteins and c-Myc: a strong connection in cancer progression. Drug Discov Today. 2020;25:891–900. 32105718 10.1016/j.drudis.2020.02.007

[38] Kim BH, Chang JH. Differential effect of GLUT1 overexpression on survival and tumor immune microenvironment of human papilloma virus type 16-positive and -negative cervical cancer. Sci Rep. 2019;9:13301. 31527827 10.1038/s41598-019-49928-x PMC6746783

[39] Riedl CC, Akhurst T, Larson S, Stanziale SF, Tuorto S, Bhargava A, et al. 18F-FDG PET scanning correlates with tissue markers of poor prognosis and predicts mortality for patients after liver resection for colorectal metastases. J Nucl Med Off Publ Soc Nucl Med. 2007;48:771–5. 10.2967/jnumed.106.037291 17475966

[40] Du D, Liu C, Qin M, Zhang X, Xi T, Yuan S, et al. Metabolic dysregulation and emerging therapeutical targets for hepatocellular carcinoma. Acta Pharm Sin B. 2022;12:558–80. 35256934 10.1016/j.apsb.2021.09.019 PMC8897153

[41] Hay N. Reprogramming glucose metabolism in cancer: can it be exploited for cancer therapy? Nat Rev Cancer. 2016;16:635–49. 27634447 10.1038/nrc.2016.77 PMC5516800

[42] Arvanitakis K, Koufakis T, Kotsa K, Germanidis G. The effects of sodium-glucose cotransporter 2 inhibitors on hepatocellular carcinoma: from molecular mechanisms to potential clinical implications. Pharmacol Res. 2022;181:106261. 35588918 10.1016/j.phrs.2022.106261

[43] Yao J, Tang S, Shi C, Lin Y, Ge L, Chen Q, et al. A potential CDK6 inhibitor, suppresses SLC2A1/GLUT1 enhancer activity to induce AMPK-ULK1-mediated cytotoxic autophagy in hepatocellular carcinoma. Autophagy. 2023;19:1221–38. 36048765 10.1080/15548627.2022.2119353 PMC10012924

[44] Chen X, Yu C, Liu X, Liu B, Wu X, Wu J, et al. Intracellular galectin-3 is a lipopolysaccharide sensor that promotes glycolysis through mTORC1 activation. Nat Commun. 2022;13:7578. 36481721 10.1038/s41467-022-35334-x PMC9732310

[45] Puris E, Fricker G, Gynther M. The role of solute carrier transporters in efficient anticancer drug delivery and therapy. Pharmaceutics. 2023;15:364. 36839686 10.3390/pharmaceutics15020364 PMC9966068

[46] Bai L, Yang ZX, Ma PF, Liu JS, Wang DS, Yu HC. Overexpression of SLC25A51 promotes hepatocellular carcinoma progression by driving aerobic glycolysis through activation of SIRT5. Free Radic Biol Med. 2022;182:11–22. 35182732 10.1016/j.freeradbiomed.2022.02.014

[47] Han X, Yang J, Li D, Guo Z. Overexpression of uric acid transporter SLC2A9 inhibits proliferation of hepatocellular carcinoma cells. Oncol Res. 2019;27:533–40. 29523220 10.3727/096504018X15199489058224 PMC7848443

[48] Koppenol WH, Bounds PL, Dang CV. Otto Warburg’s contributions to current concepts of cancer metabolism. Nat Rev Cancer. 2011;11:325–37. 21508971 10.1038/nrc3038

[49] San-Millán I, Brooks GA. Reexamining cancer metabolism: lactate production for carcinogenesis could be the purpose and explanation of the Warburg Effect. Carcinogenesis. 2017;38:119–33. 27993896 10.1093/carcin/bgw127 PMC5862360

[50] Zhou Y, Lin F, Wan T, Chen A, Wang H, Jiang B, et al. ZEB1 enhances Warburg effect to facilitate tumorigenesis and metastasis of HCC by transcriptionally activating PFKM. Theranostics. 2021;11:5926–38. 33897890 10.7150/thno.56490 PMC8058737

[51] Aft RL, Zhang FW, Gius D. Evaluation of 2-deoxy-D-glucose as a chemotherapeutic agent: mechanism of cell death. Br J cancer. 2002;87:805–12. 12232767 10.1038/sj.bjc.6600547 PMC2364258

[52] Zhang Y, Huang F, Wang J, Luo H, Wang Z. 2-DG-regulated RIP and c-FLIP effect on liver cancer cell apoptosis induced by TRAIL. Med Sci Monit Int Med J Exp Clin Res. 2015;21:3442–8. 10.12659/MSM.895034 PMC4646230 26552967

[53] Tesori V, Piscaglia AC, Samengo D, Barba M, Bernardini C, Scatena R, et al. The multikinase inhibitor Sorafenib enhances glycolysis and synergizes with glycolysis blockade for cancer cell killing. Sci Rep. 2015;5:9149. 25779766 10.1038/srep09149 PMC4361992

[54] Cadet J, Davies KJA. Oxidative DNA damage & repair: an introduction. Free Radic Biol Med. 2017;107:2–12. 28363603 10.1016/j.freeradbiomed.2017.03.030 PMC5510741

[55] Gilliam LA, Moylan JS, Patterson EW, Smith JD, Wilson AS, Rabbani Z, et al. Doxorubicin acts via mitochondrial ROS to stimulate catabolism in C2C12 myotubes. Am J Physiol Cell Physiol. 2012;302:C195–202. 21940668 10.1152/ajpcell.00217.2011 PMC3328915

[56] Kuczler MD, Olseen AM, Pienta KJ, Amend SR. ROS-induced cell cycle arrest as a mechanism of resistance in polyaneuploid cancer cells (PACCs). Prog Biophys Mol Biol. 2021;165:3–7. 33991583 10.1016/j.pbiomolbio.2021.05.002 PMC8511226

[57] Benkafadar N, François F, Affortit C, Casas F, Ceccato JC, Menardo J, et al. ROS-induced activation of DNA damage responses drives senescence-like state in postmitotic cochlear cells: implication for hearing preservation. Mol Neurobiol. 2019;56:5950–69. 30693443 10.1007/s12035-019-1493-6 PMC6614136

[58] Liu J, Harada BT, He C. Regulation of gene expression by N(6)-methyladenosine in cancer. Trends Cell Biol. 2019;29:487–99. 30940398 10.1016/j.tcb.2019.02.008 PMC6527461

[59] Xia P, Zhang H, Lu H, Xu K, Jiang X, Jiang Y, et al. METTL5 stabilizes c-Myc by facilitating USP5 translation to reprogram glucose metabolism and promote hepatocellular carcinoma progression. Cancer Commun. 2023;43:338–64. 10.1002/cac2.12403 PMC10009668 36602428

[60] Lin Y, Wei X, Jian Z, Zhang X. METTL3 expression is associated with glycolysis metabolism and sensitivity to glycolytic stress in hepatocellular carcinoma. Cancer Med. 2020;9:2859–67. 32068977 10.1002/cam4.2918 PMC7163109

[61] Li S, Wu Q, Liu J, Zhong Y. Identification of two m6A readers YTHDF1 and IGF2BP2 as immune biomarkers in head and neck squamous cell carcinoma. Front Genet. 2022;13:903634. 35646049 10.3389/fgene.2022.903634 PMC9133459

[62] Zhang Z, Xing Y, Gao W, Yang L, Shi J, Song W, et al. N(6)-methyladenosine (m(6)A) reader IGF2BP2 promotes gastric cancer progression via targeting SIRT1. Bioengineered. 2022;13:11541–50. 35502827 10.1080/21655979.2022.2068920 PMC9275927

[63] Yao B, Zhang Q, Yang Z, An F, Nie H, Wang H, et al. CircEZH2/miR-133b/IGF2BP2 aggravates colorectal cancer progression via enhancing the stability of m(6)A-modified CREB1 mRNA. Mol Cancer. 2022;21:140. 35773744 10.1186/s12943-022-01608-7 PMC9245290

[64] Liu Y, Shi M, He X, Cao Y, Liu P, Li F, et al. LncRNA-PACERR induces pro-tumour macrophages via interacting with miR-671-3p and m6A-reader IGF2BP2 in pancreatic ductal adenocarcinoma. J Hematol Oncol. 2022;15:52. 35526050 10.1186/s13045-022-01272-w PMC9077921

[65] Chan TK, Loh XY, Peh HY, Tan WNF, Tan WSD, Li N, et al. House dust mite-induced asthma causes oxidative damage and DNA double-strand breaks in the lungs. J Allergy Clin Immunol. 2016;138:84–96. 27157131 10.1016/j.jaci.2016.02.017

