# Supplementary material for: SLC50A1 inhibits the doxorubicin sensitivity in hepatocellular carcinoma cells through regulating the tumor glycolysis
Source: Cell Death Discov. 2024 Dec 18;10:495. doi: 10.1038/s41420-024-02261-3 (PMC11655560; doi:10.1038/s41420-024-02261-3)
Supplement: Supplementary file 1 — SUPPLEMENTAL MATERIAL [file 41420_2024_2261_MOESM1_ESM.docx]

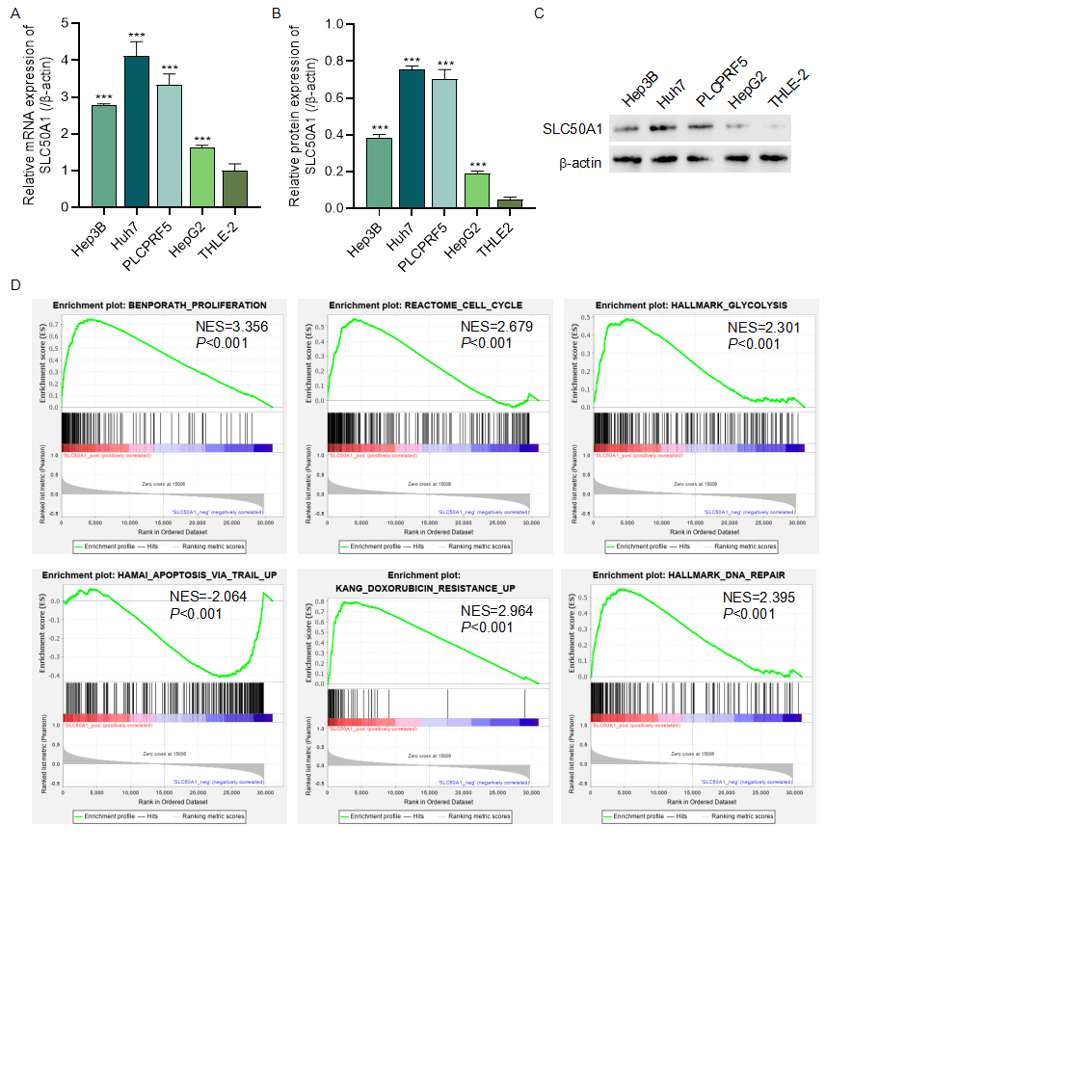


Figure S1. SLC50A1 expression in HCC cell lines and correlates the signaling pathways. (A-C) The relative mRNA and protein levels of SLC50A1 in various HCC cell lines (Hep3B, HuH7, PLCPRF5, and HepG2) and human healthy hepatocytes (THLE-2 cells). (D) GESA demonstrated that SLC50A1 expression was associated with BENPORATH_PROLIFERATION, REACTOME_CELL_CYCLE, HALLMARK_GLYCOLYSIS, HAMAI_APOPTOSIS_VIA_TRAIL_UP, KANG_DOXORUBICIN_RESISTANCE_UP, and HALLMARK_DNA_REPAIR pathways. ***P<0.001 vs THLE-2 group.


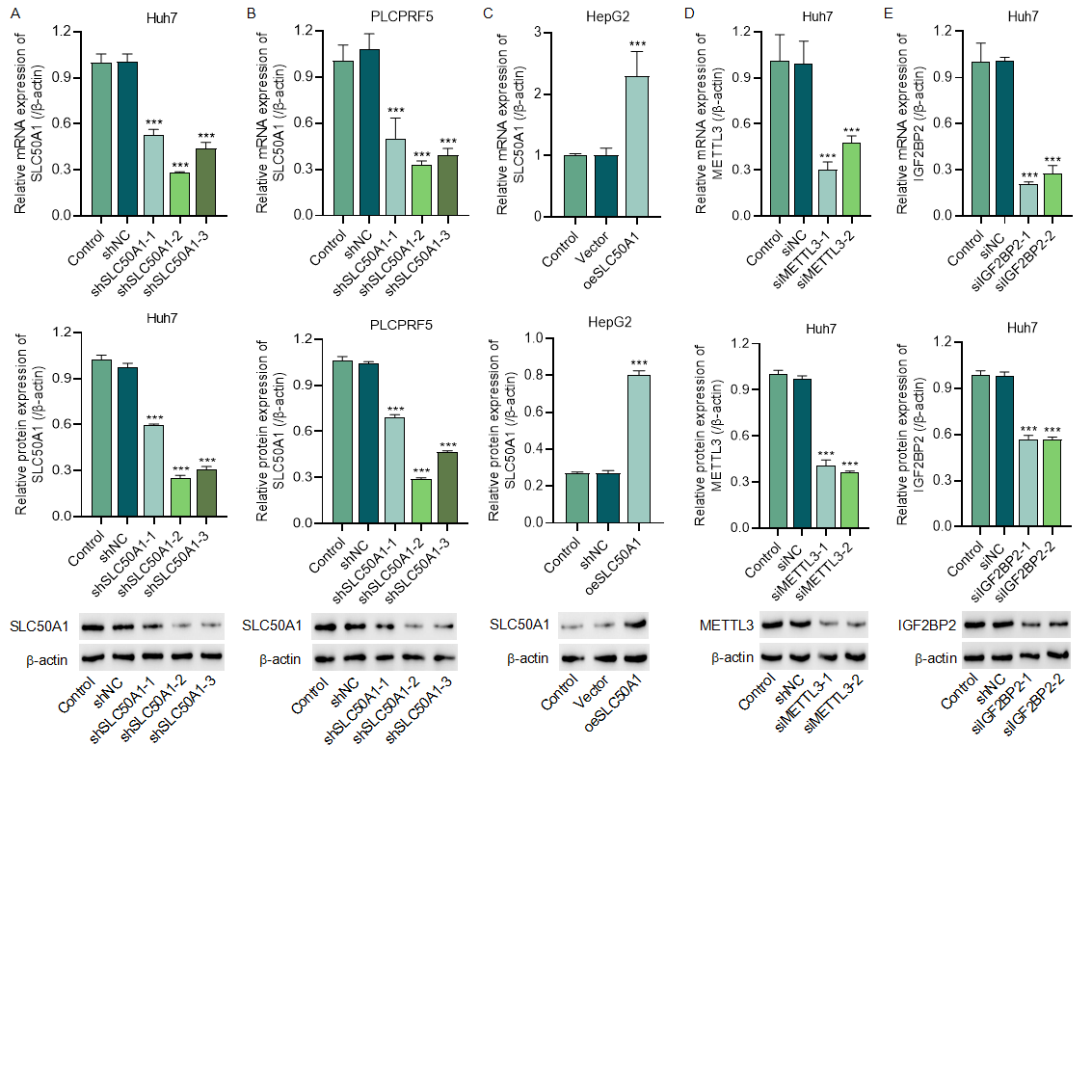


Figure S2. Knockdown and overexpression in HCC cell lines. SLC50A1 mRNA and protein expression in (A) Huh7 and (C) PLCPRF5 cells with SLC50A1 knockdown or in (C) HepG2 cells with SLC50A1 overexpression. (D) METTL3 or (E) IGF2BP2 mRNA and protein expression in Huh7 cells with METTL3 or IGF2BP2 knockdown. ***P<0.001 vs shNC, vector or siNC group.


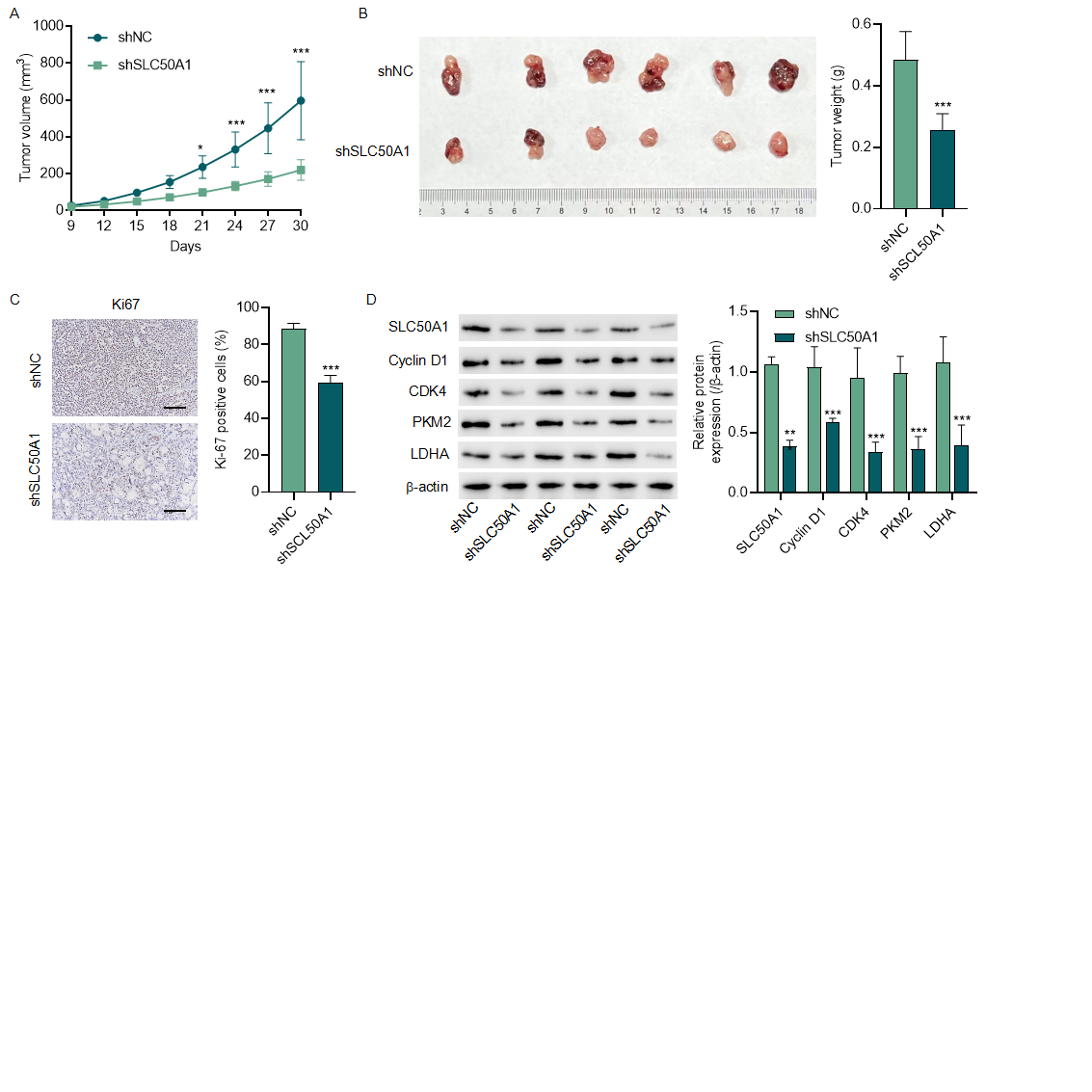


Figure S3. SLC50A1 knockdown inhibits tumor growth in vivo. Huh7 cells transduced with shNC or SLC50A1 shRNA vector were subcutaneously injected into the armpits of the nude mice. (A) Tumor volume was monitored every 3 days for 30 days. (B) At day 30, mice were sacrificed, and tumors were photographed and weighed. (C) Ki67 IHC staining. (D) Expression of SLC50A1, Cyclin D1, CDK4, PKM2 and LDHA. Scale bar: 100 μm. *P<0.05, ***P<0.001 vs shNC group.


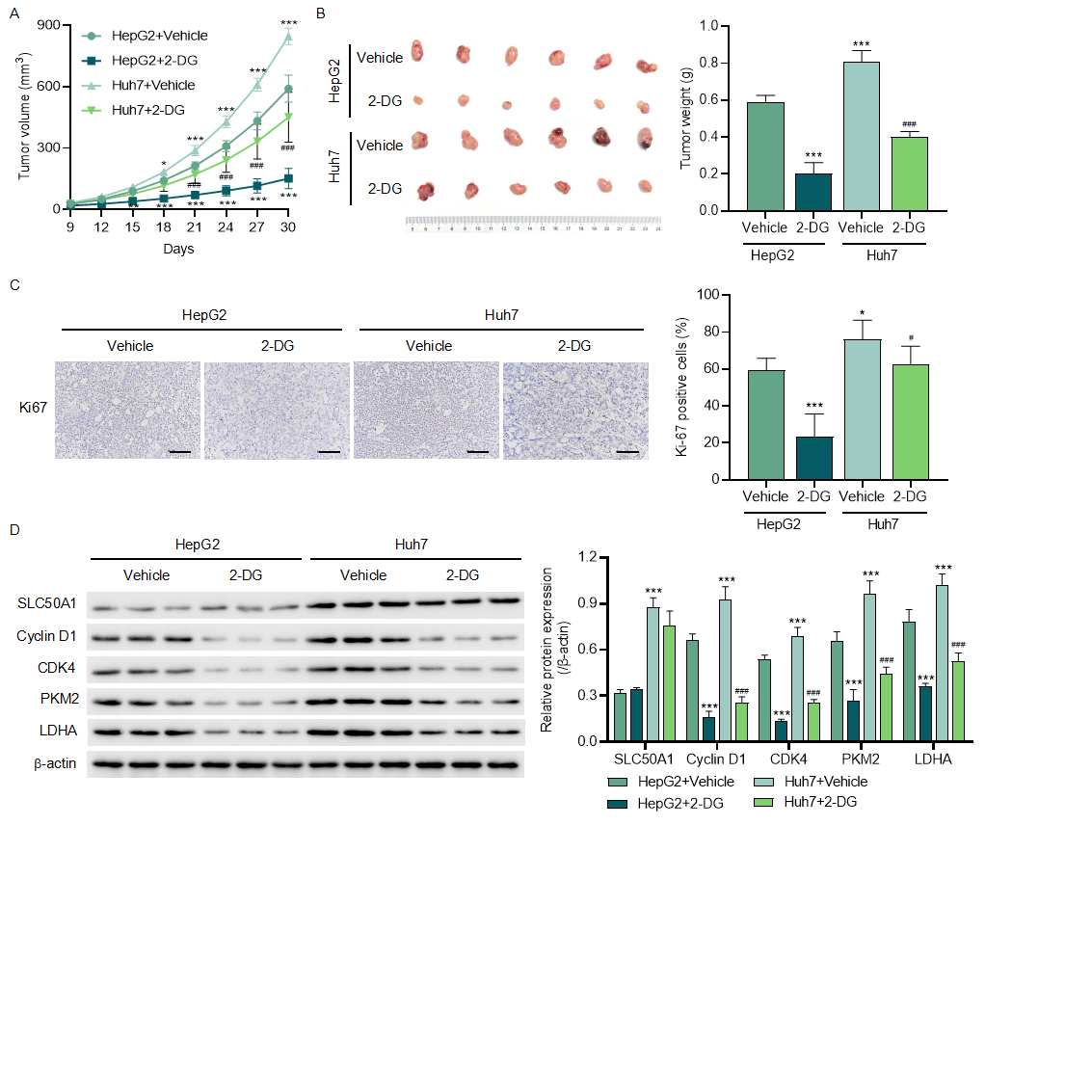


Figure S4. 2-DG inhibits tumor growth in vivo. Huh7 or HepG2 cells were subcutaneously injected into the armpits of the nude mice with or without 2-DG treatment. (A) Tumor volume was monitored every 3 days for 30 days. (B) At day 30, mice were sacrificed, and tumors were photographed and weighed. (C) Ki67 IHC staining. (D) Expression of SLC50A1, Cyclin D1, CDK4, PKM2 and LDHA. Scale bar: 100 μm. *P<0.05, ***P<0.001 vs HepG2 (Vehicle) group. #P<0.05, ###P<0.001 vs Huh7 (Vehicle) group.


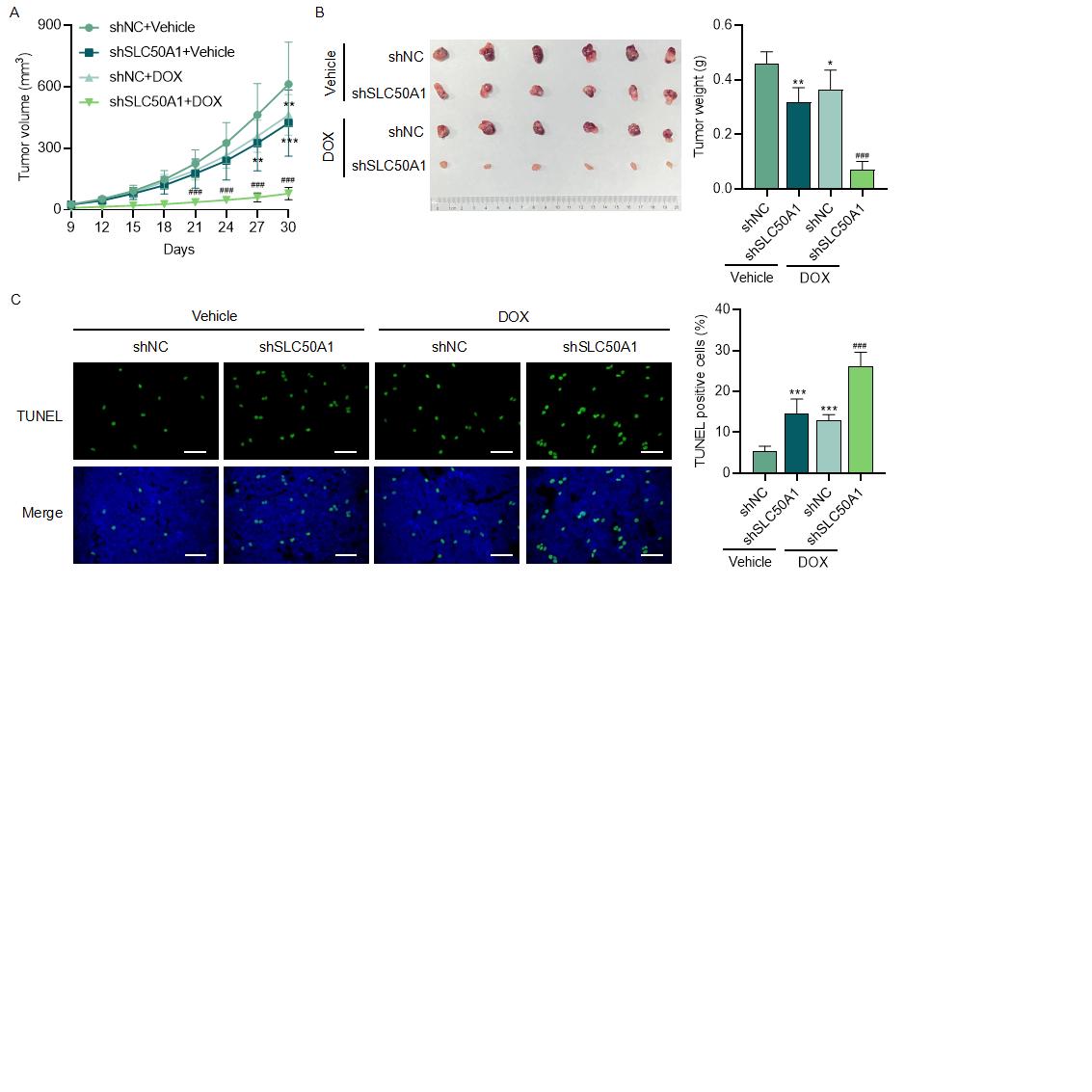


Figure S5. SLC50A1 knockdown promotes the DOX sensitivity in HCC in vivo. Huh7 cells transduced with SLC50A1 shRNA vector were subcutaneously injected into the armpits of the nude mice with or without DOX treatment. (A) Tumor volume was monitored every 3 days for 30 days. (B) At day 30, mice were sacrificed, and tumors were photographed and weighed. (C) TUNEL staining. Scale bar: 100 μm. *P<0.05, **P<0.01, ***P<0.001 vs shNC+Vehicle group. ###P<0.001 vs shNC+DOX group.
