# Supplementary material for: SLC50A1 inhibits the doxorubicin sensitivity in hepatocellular carcinoma cells through regulating the tumor glycolysis
Source: Cell Death Discov. 2024 Dec 18;10:495. doi: 10.1038/s41420-024-02261-3 (PMC11655560; doi:10.1038/s41420-024-02261-3)

Figure 1I


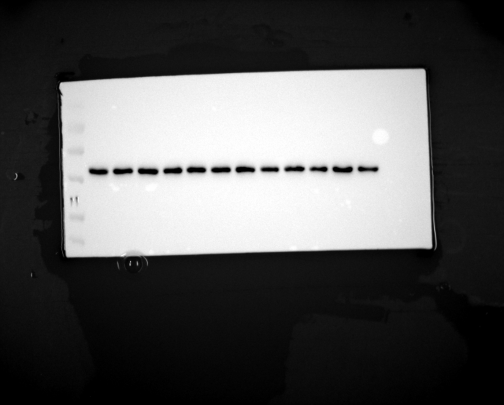

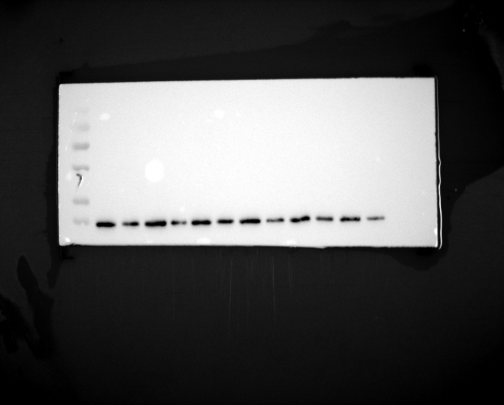


Figure 2H

HUH7


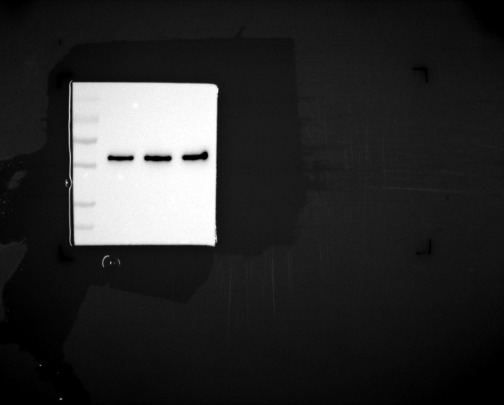

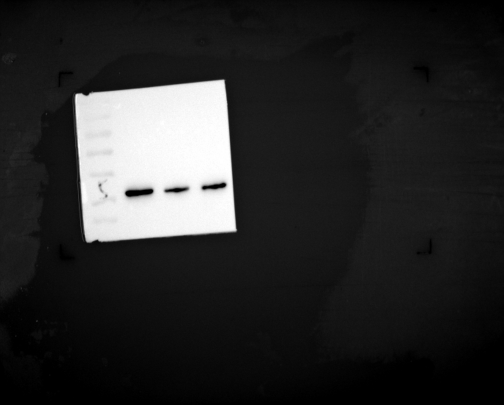

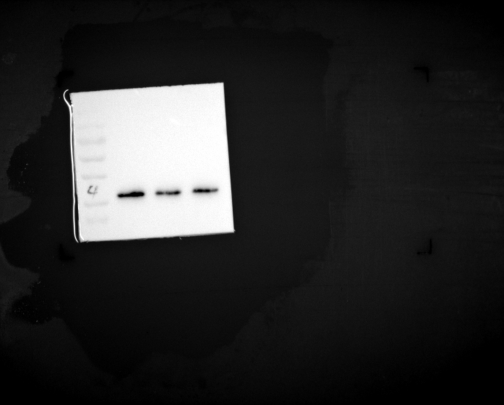


PLCPRF5


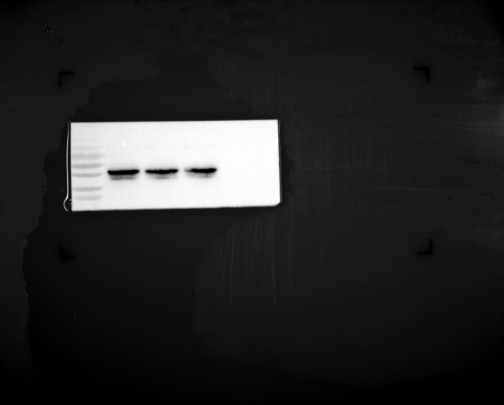

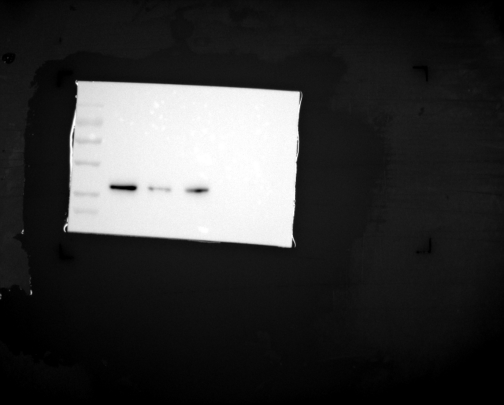

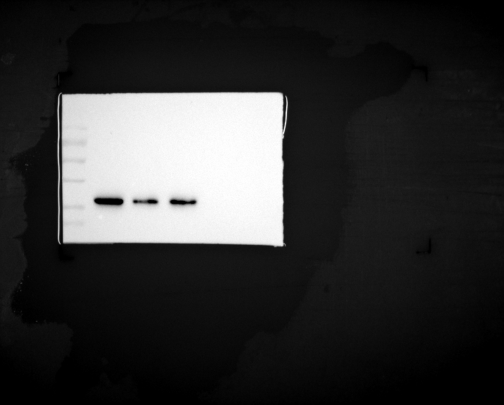


Figure 3G

HUH7


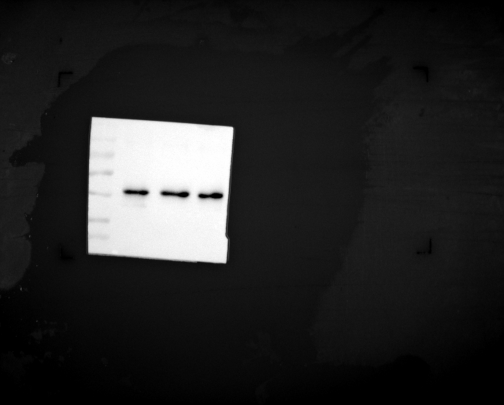

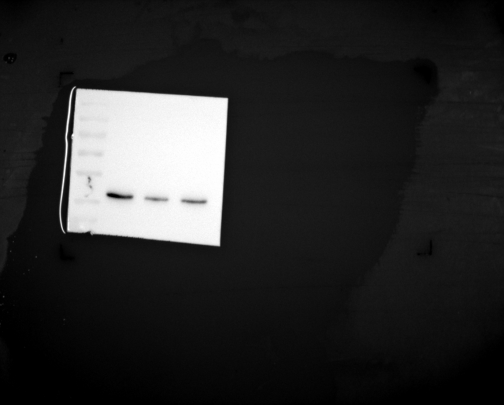

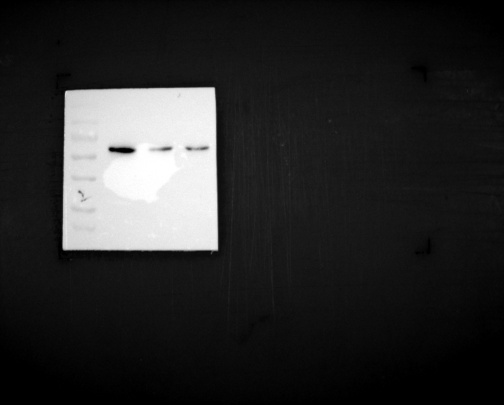


PLCPRF5


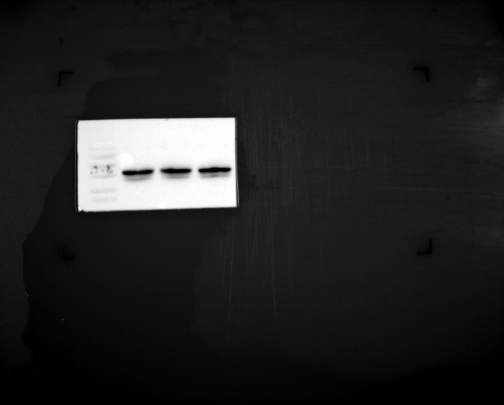

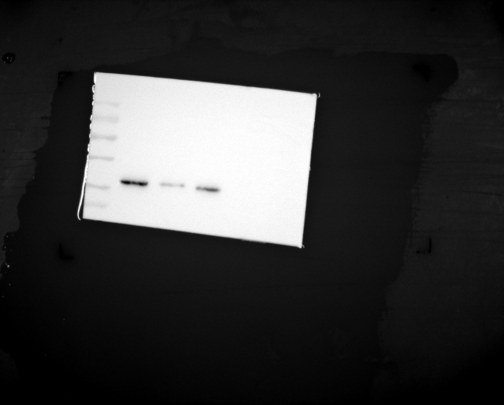

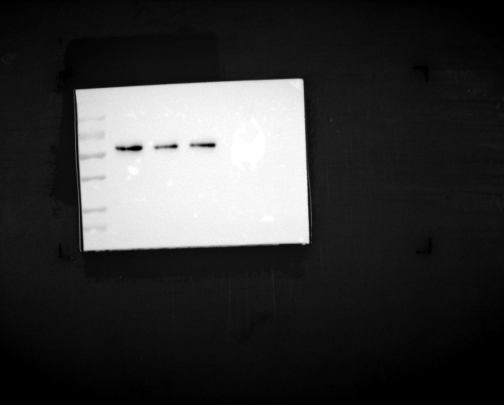


Figure 4H


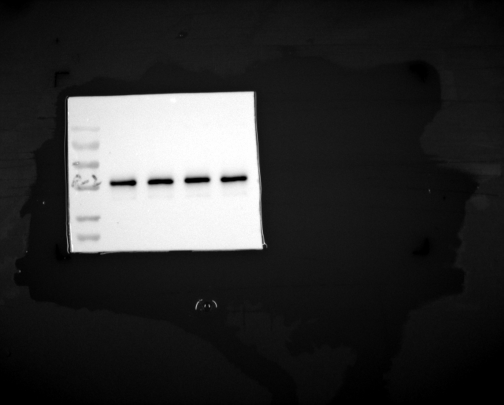

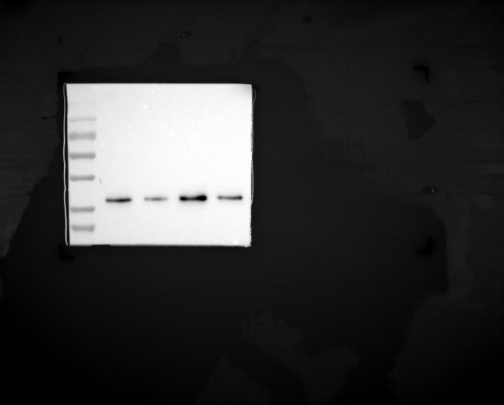

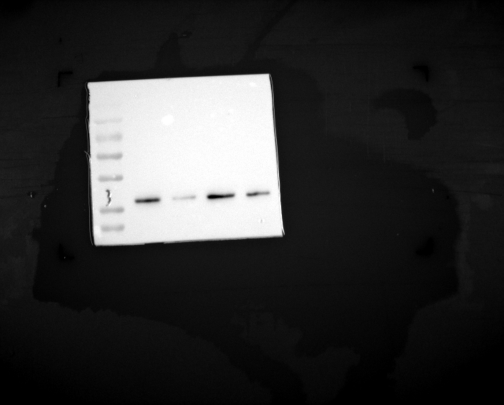

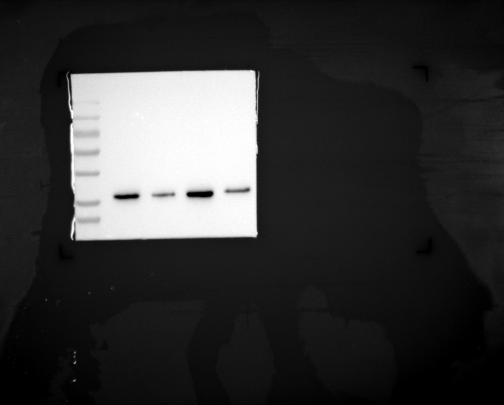

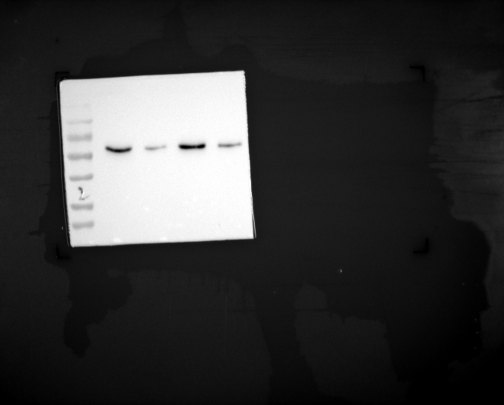


Figure 5F


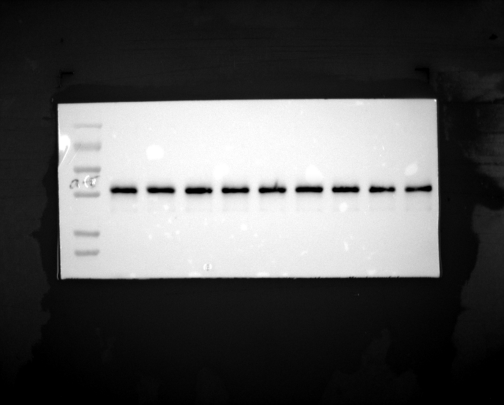

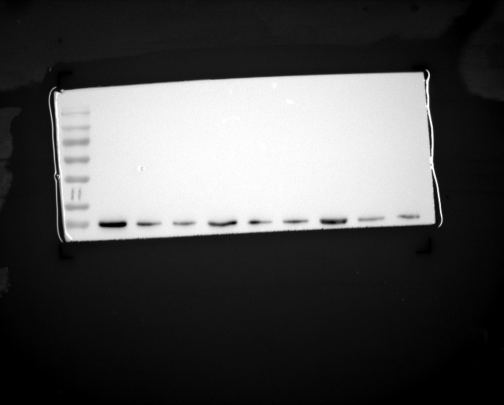


Figure 7G


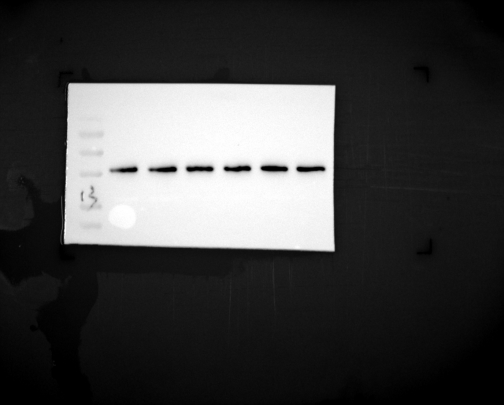

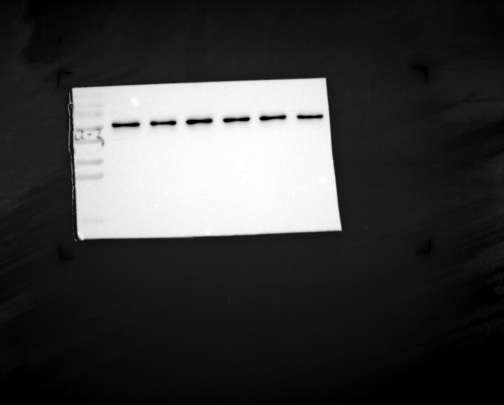

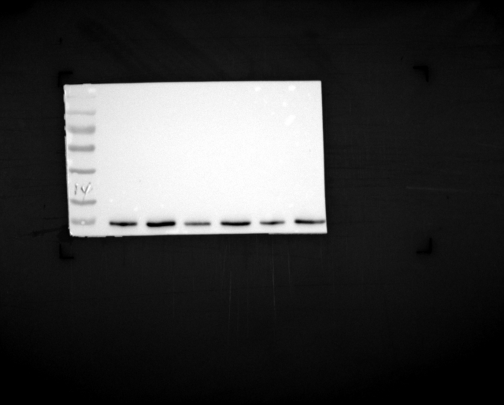

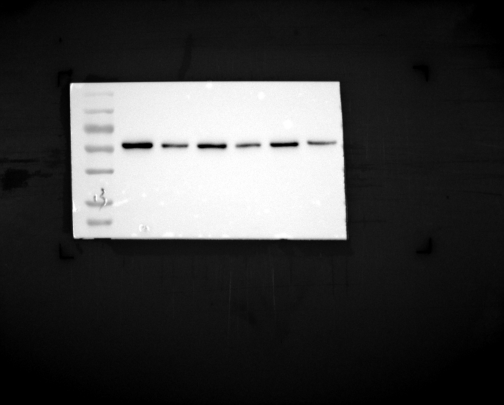


Figure 7H


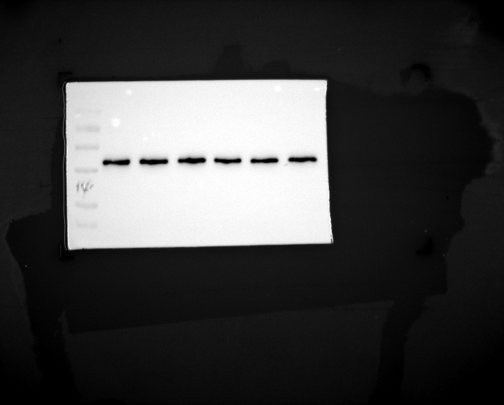

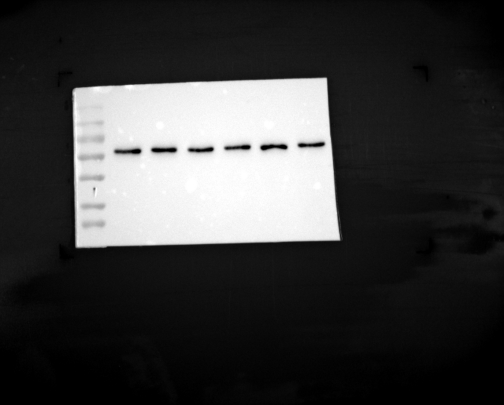

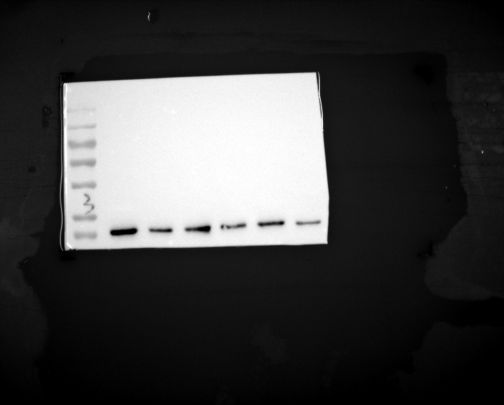

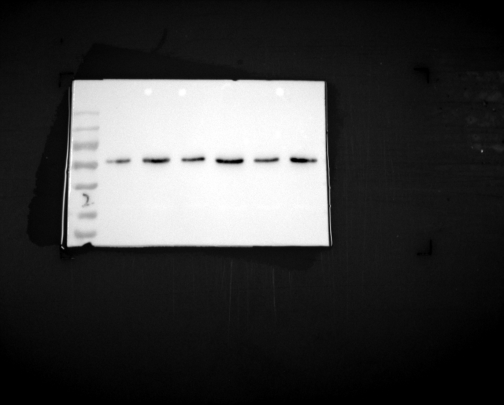


Figure 8E


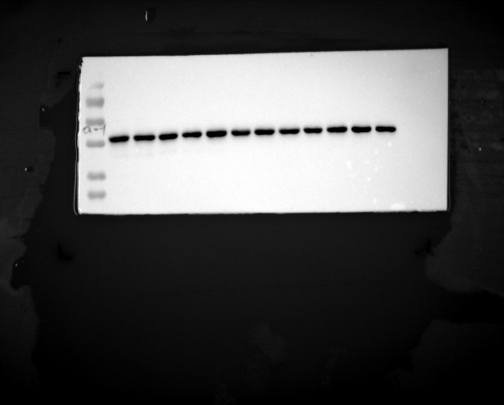

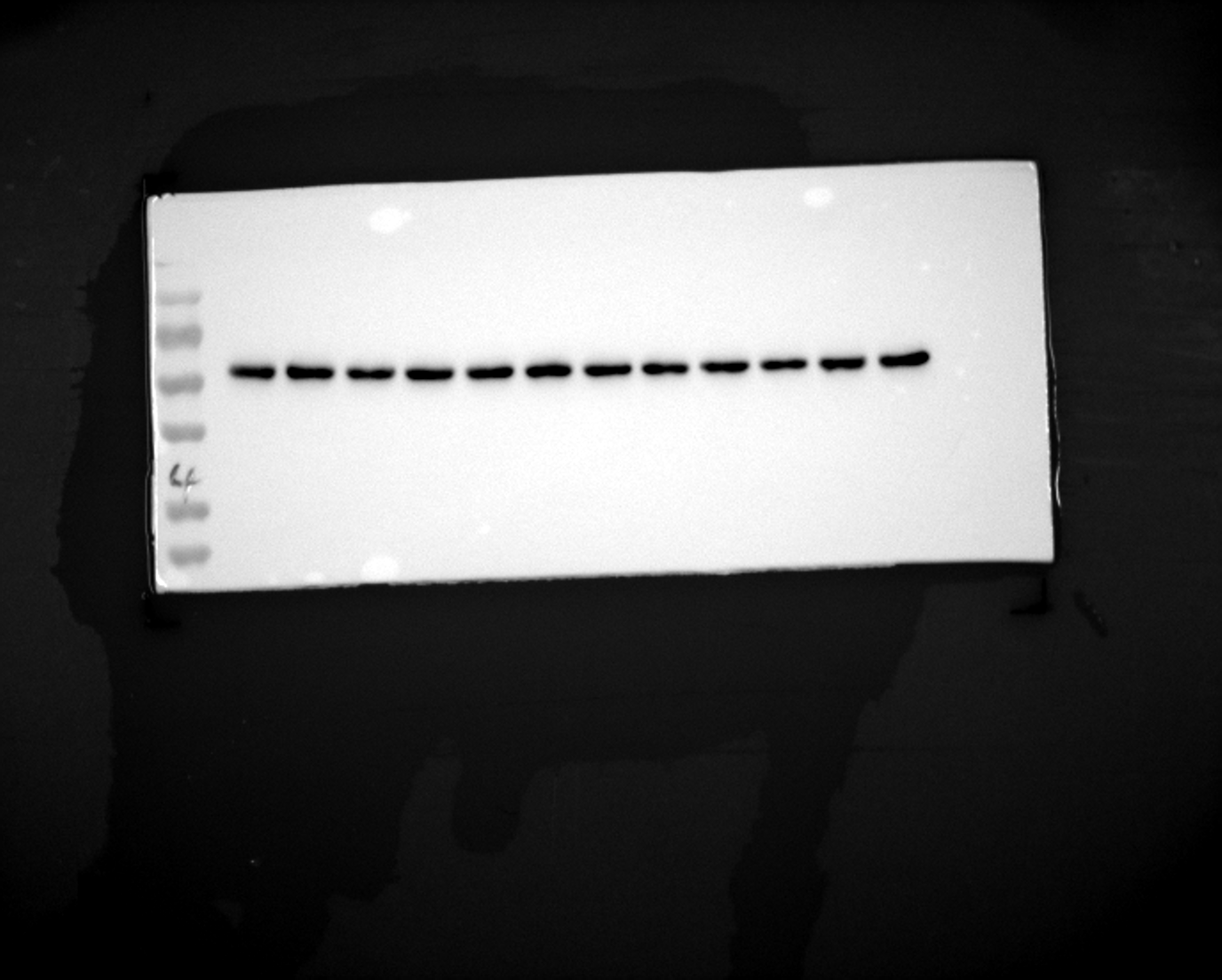

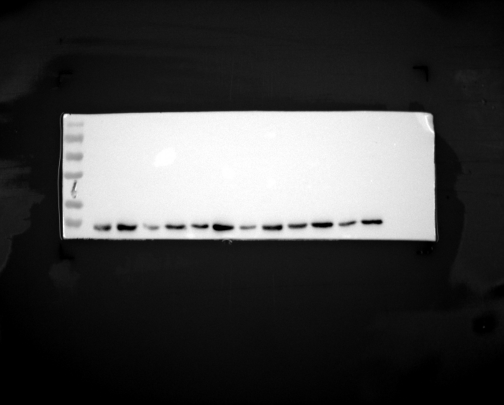

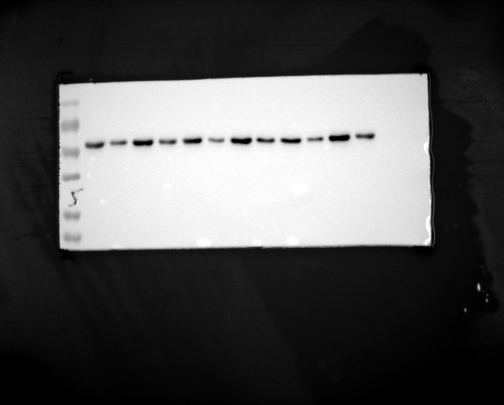


Figure S1C


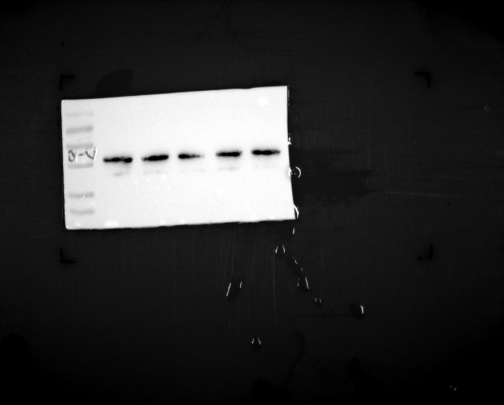

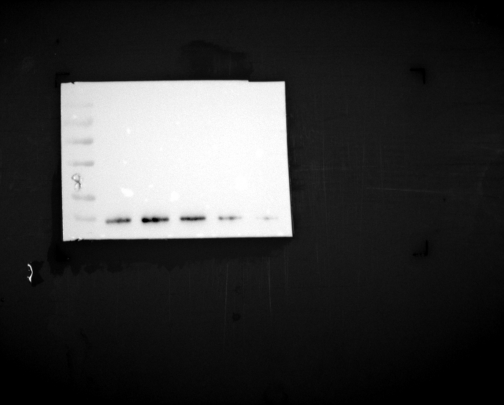


Figure S2A


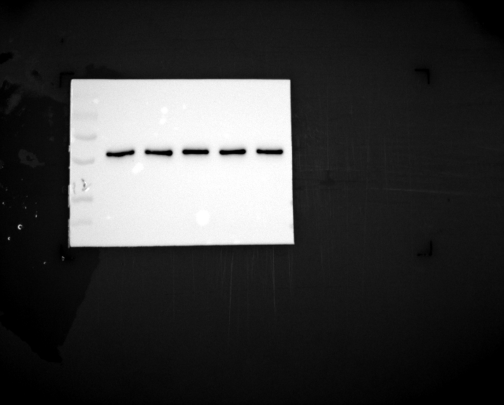

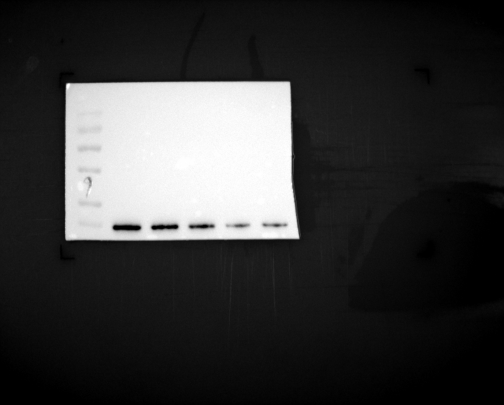


Figure S2B


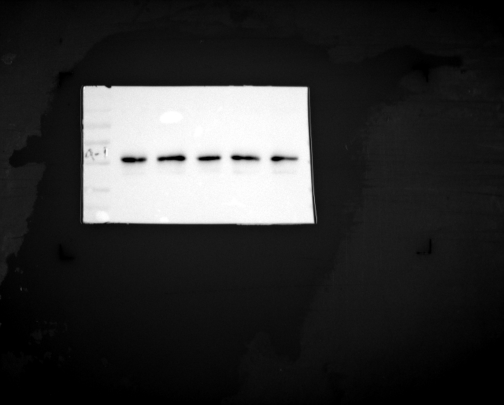

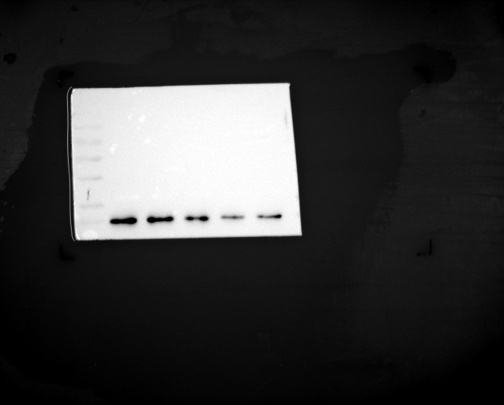


Figure S2C


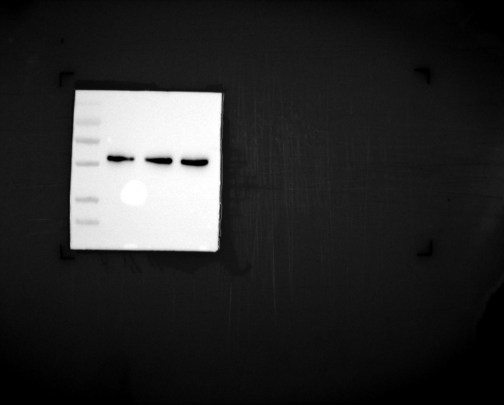

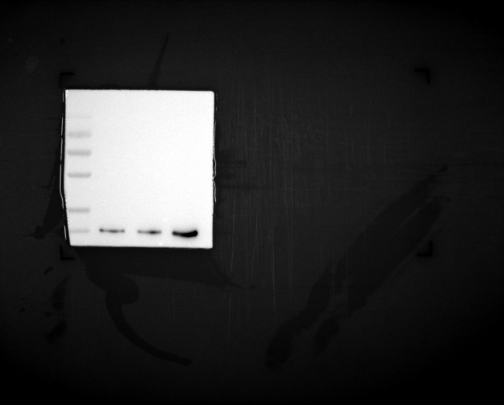


Figure S2D


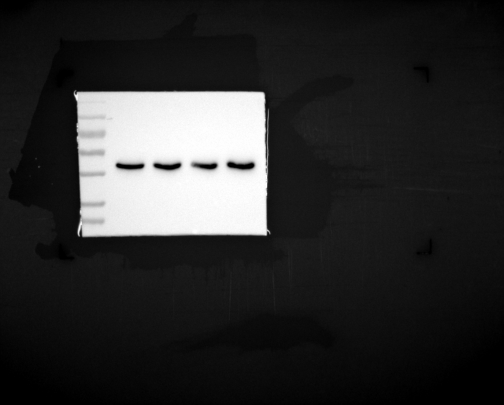

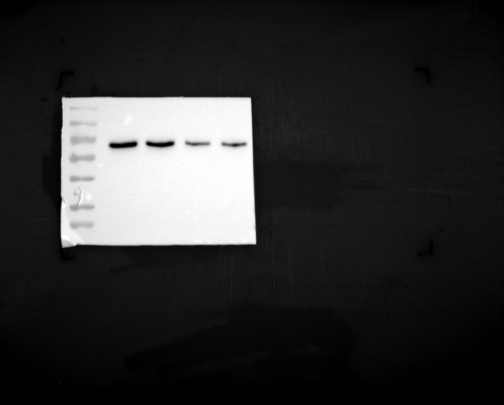


Figure S2E


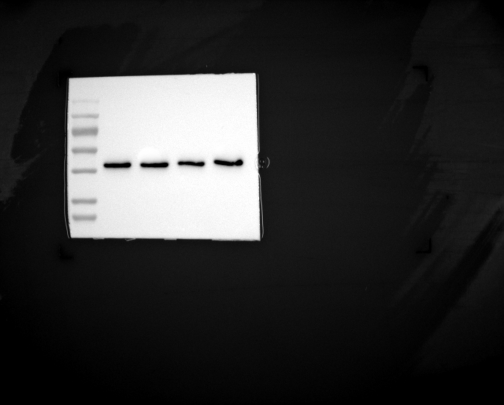

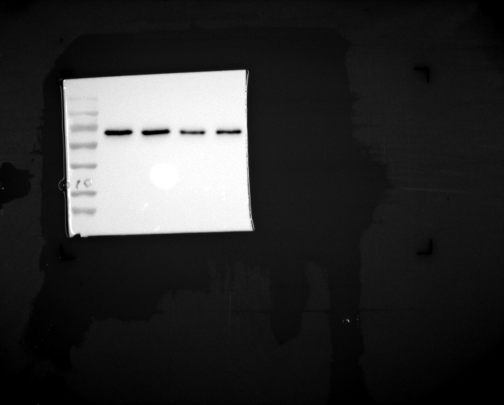


Figure S3D


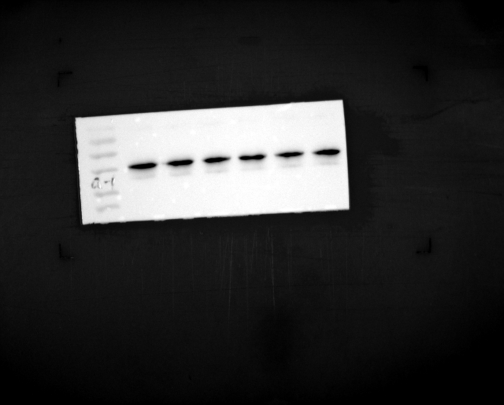

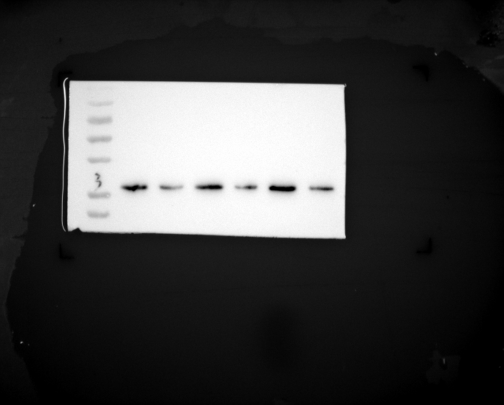

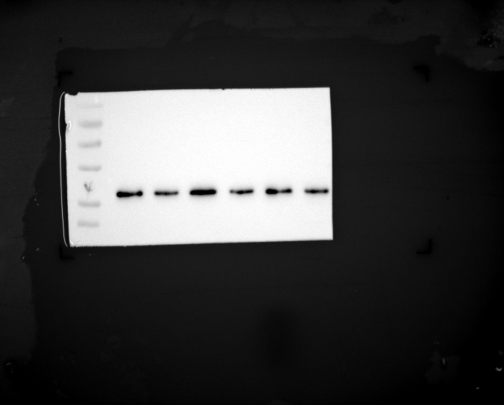

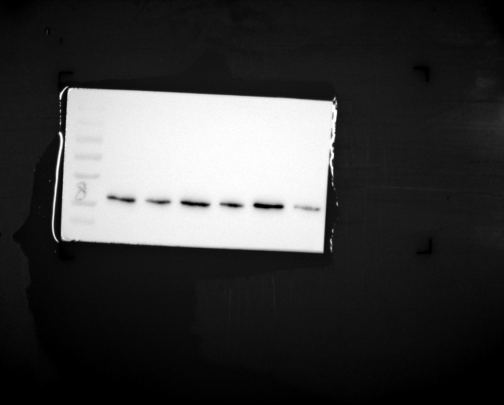

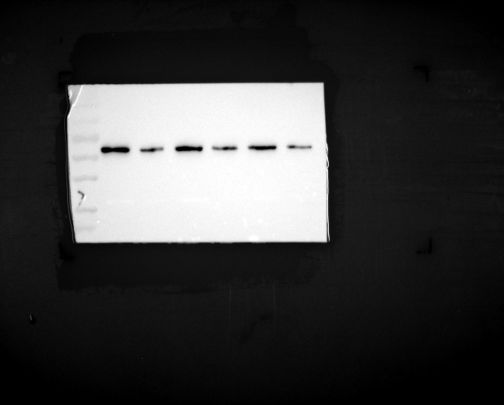

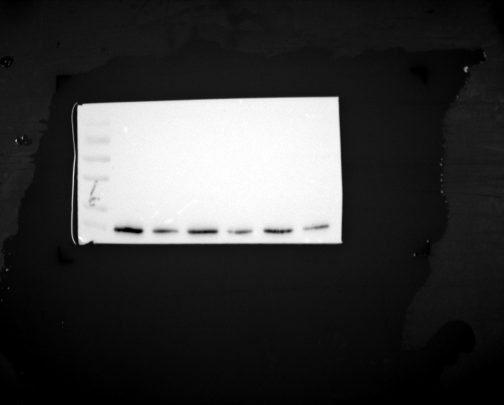


Figure S4D


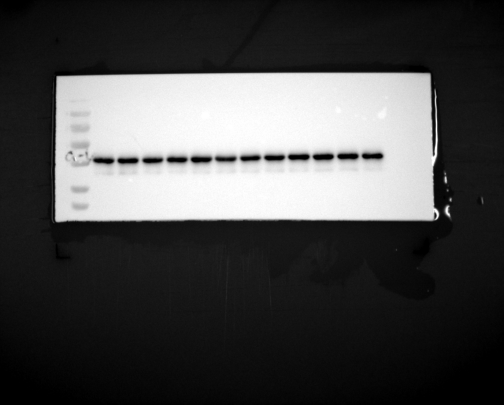

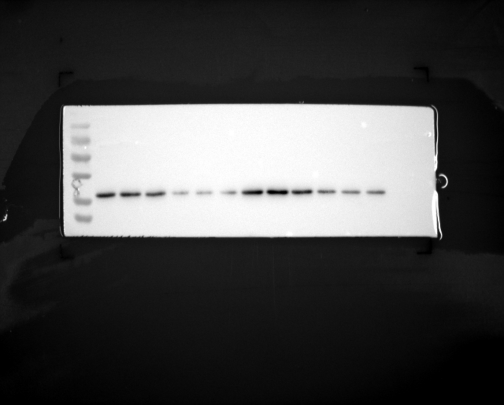

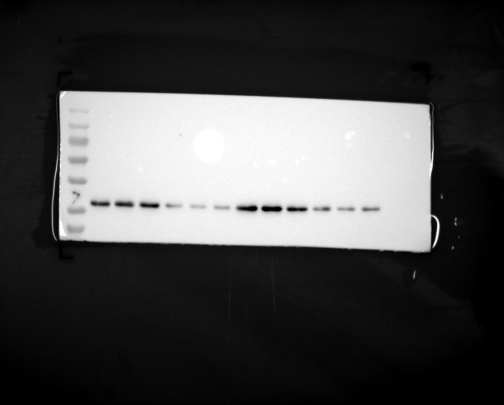

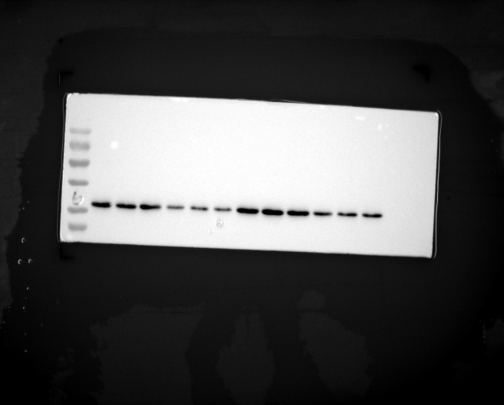

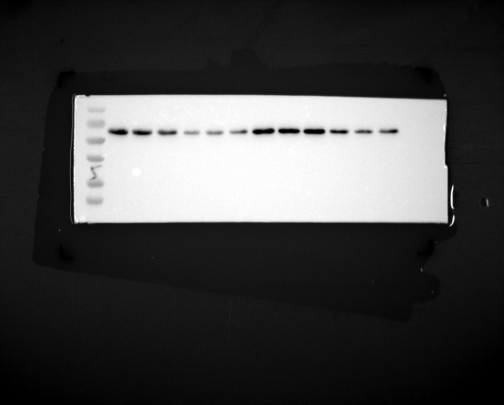

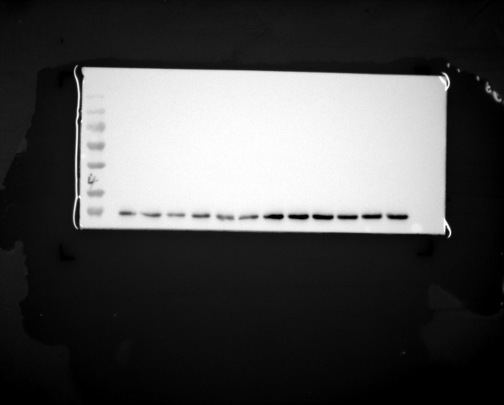

Supplement: Supplementary file 2 — Original Data File [file 41420_2024_2261_MOESM2_ESM.docx]
